# Comparative electrothermal analysis of single and small-electrode grid configurations for HD-inspired peripheral transcutaneous neurostimulation

**DOI:** 10.3389/fbioe.2026.1904885

**Published:** 2026-08-28

**Authors:** Yuanshan Zhong, Filip Stefanovic

**Affiliations:** Department of Biomedical Engineering, School of Engineering and Applied Sciences, University at Buffalo, Buffalo, NY, United States

**Keywords:** current density, electrode subdivision, electrothermal behavior, electrothermal modeling, finite element method, high-definition electrode, peripheral transcutaneous stimulation, surface neurostimulation

## Abstract

**Background:**

High-density (HD)-inspired peripheral transcutaneous neurostimulation systems employing clusters of small-area electrode arrays may improve spatial controllability during electrical stimulation of superficial limb tissues, as used in TENS/NMES/FES-like wearable applications. However, the electrothermal consequences of small electrodes can affect usability due to complexities in safety considerations. Therefore it is of critical interest to understand the effects of these systems, and to determine how subdivision topology shapes localized current concentration and thermal accumulation under fixed electrode geometry conditions.

**Objective:**

This study investigates how high-density clusters of small-area electrodes influences current-density distribution and electrothermal behavior. This project is part of a larger study to design and optimize these high-density neurostimulation arrays to improve spatial scaling.

**Methods:**

A coupled electrical–thermal finite-element model was developed in COMSOL Multiphysics 6.3 to compare conventional single-electrode configurations with a systematic series of HD-inspired small-electrode grid configurations ranging from 1 to 16 electrodes (grid1 through grid16). The individual electrode radius and inter-electrode spacing were held constant while the number of active electrodes was varied. Current-density distribution, temperature change, and thermal transient effects were evaluated using current-controlled pulsed stimulation with varying current amplitudes, pulse widths, and frequencies.

**Results:**

Electrothermal effects did not increase linearly with the number of active electrodes. Three distinct electrothermal regimes were identified based on stimulation-side behavior: an extreme concentration regime ( N=1 –4), a transition regime ( N=5 –8), and a regime in which stimulation-side load falls below a fixed return-electrode background contribution ( N≥9 , crossover $N^{*}=8$ –9), where N is the number of active electrodes in a grid. The N=9 regime showed the closest stimulation-side electrothermal behavior to the single-electrode reference among all configurations tested ( ΔT=1.340 °C vs. 1.370 °C, or a difference of 0.030 °C). The stimulation-side ΔT declined with electrode count across this full range ( $\Delta T\approx 9.69\times N^{-0.91}$ , $R^{2}=0.999$ ). A dedicated area-matched control series, in which electrode radius was increased to hold total active area constant showed a decline in stimulation-side current density and a temperature rise with electrode count. A complementary, exploratory comparison holding electrode count fixed while varying the total active area also showed decline in both quantities. Together, these results indicate that electrode count and total active area each contribute to the observed electrothermal decline. However, causal effects were not isolated from one another. As expected from established current-concentration effects of small electrodes, the sparse grid1 configuration produced the highest superficial current density and temperature rise among the investigated configurations ( ΔT=10.18 °C, or 7.43 × reference-electrode baseline). A discretized parameter-space analysis (128 sampled current amplitude/pulse-width/frequency combinations) showed that the ΔT≥8 °C region occupied by each configuration declined continuously with electrode count. Specifically, from 72 combinations for grid1 (4.50 × single) to 4 combinations for grid16 (0.25 × single). Therefore, electrode grid configurations with higher electrode counts distribute the current across a progressively larger area, and thus reduced per-electrode current density.

**Conclusion:**

Under the idealized modeling conditions investigated here, electrothermal behavior was jointly determined by electrode count and total active electrode area. These two variables are intrinsically coupled in the fixed-radius grid1–grid16 design where both contribute to a reduction in stimulation-side electrothermal load. These findings represent comparative, geometry-specific modeling trends rather than validated thermal safety predictions. The present study provides a comparative computational framework for evaluating HD-inspired electrode subdivision strategies in compact peripheral transcutaneous neurostimulation design, where electrode count and total active area jointly determine the resulting current-distribution topology and associated localized thermal accumulation.

## 1 Introduction

Skin surface electrical neurostimulation is widely used in rehabilitation, neuromodulation, pain management, and wearable bioelectronic systems because it enables a non-invasive neural activation through an externally applied interface (Johnson, 2007; Liu et al., 2024; RaviChandran et al., 2023). Previous studies spanning both transcranial (e.g., HD-tDCS) and transcutaneous electrical stimulation have demonstrated that the electrode-tissue interface strongly influences electric field distribution, current density concentration, stimulation focality, and user comfort (RaviChandran et al., 2020; Datta et al., 2008; Dmochowski et al., 2011; Saturnino et al., 2015); these general electrode-interface principles motivate the present work, although the transcranial studies cited here model head-specific anatomy (skull, cerebrospinal fluid, gray and white matter) not represented in the present limb-like model, and their quantitative findings on field focality should not be assumed to transfer directly to the peripheral transcutaneous configuration investigated here. In particular, the electrode geometry and active electrode area play important roles in shaping stimulation behavior and localized current concentration within the superficial biological tissues (RaviChandran et al. 2020; Datta et al., 2008).

Conventional surface stimulation systems commonly employ relatively large electrodes to provide stable skin contact and broad current delivery. Notably, the larger electrode sizes have implications on safety due to low charge density at the interface, and better user comfort. However, these electrodes also diffuse the active area, leading to reduced spatial selectivity, limiting the ability to achieve a highly localized stimulation (Dmochowski et al., 2011; Saturnino et al., 2015). These limitations have motivated increasing interest in clustered small-electrode configurations that may provide improved spatial controllability and greater flexibility in current delivery while remaining compatible with compact and wearable neurostimulation systems (Liu et al., 2024; RaviChandran et al., 2023).

To improve stimulation selectivity and electric field controllability, recent neurostimulation studies have explored small-electrode and dense-array stimulation architectures (Villamar et al., 2013; Datta et al., 2009; Guler et al., 2016; Jain et al., 2023). For example, Malešević et al. (2012) demonstrated a multi-pad functional electrical stimulation system employing an array of small electrode contacts (each approximately 1–4 mm in diameter, spaced several millimeters apart) to achieve selective forearm muscle activation, showing that spatially distributed small-contact electrode arrays can improve current steering in transcutaneous applications—a peripheral stimulation context directly relevant to the present model. Similarly, Villamar et al. (2013) described a 4×1 ring HD-tDCS configuration in which a central electrode (radius ≈ 6 mm) was surrounded by four return electrodes at a fixed spacing, demonstrating improved focality over conventional pad electrodes; this and related HD-tDCS focality findings were obtained in a transcranial head model and are cited here only as general background on clustered-electrode current steering, not as directly transferable predictions for the peripheral limb geometry investigated in the present study. In high-definition electrical stimulation systems, clustered electrode layouts and multichannel electrode arrangements have been shown to improve spatial targeting and current steering capability compared with conventional large-pad stimulation approaches (Datta et al., 2009; Guler et al., 2016). Computational studies have further demonstrated that electrode geometry and active electrode distribution substantially influence local electric field patterns and current density concentration within superficial tissues (Datta et al., 2009; Guler et al., 2016; Jain et al., 2023; Mancabelli et al., 2026).

The HD electrode design philosophy—replacing a conventional large stimulation surface with an array of smaller electrode elements—is relevant beyond transcranial applications, extending to wearable and compact surface neurostimulation systems where distributed electrode interfaces may provide greater flexibility in current routing and adaptive stimulation control (Liu et al., 2024; RaviChandran et al., 2023). The present study adopts this HD design approach by using arrays of small electrodes (radius 1 mm, center-to-center spacing 3 mm) arranged within a single stimulation pole. It is important to clarify precisely what “HD-inspired” denotes in the present context: the small-electrode grid configurations investigated here are treated as a single stimulation pole with the total current allocated equally across active contacts (Section 2.3.2), not as an optimized multichannel current-steering array of the kind used in conventional HD-tDCS montages (e.g., 4×1 ring configurations with weighted return electrodes (Villamar et al., 2013) or in gyri-precise, field-shaping HD electrode designs (Datta et al., 2009; Guler et al., 2016). The term “HD-inspired” is used here to denote the underlying design philosophy of electrode subdivision within a compact footprint, rather than equivalence to any specific conventional HD-tDCS or current-steered multichannel architecture.

However, increasing the number of clustered electrodes within a stimulation interface simultaneously modifies effective active area and local current crowding behavior near the electrode-tissue interface. Therefore, the electrothermal consequences of electrode cluster numbers cannot be assumed to scale with electrode count, and a systematic investigation is required to characterize how electrode topology governs current concentration and thermal accumulation under fixed electrode geometry conditions.

Electrothermal behavior remains an important consideration in surface neurostimulation due to heating near the electrode-tissue interface. In transcranial stimulation—modeled with head-specific anatomy including skull, CSF, and gray/white matter—as well as in transcutaneous electrical stimulation and bioelectronic skin interfaces more directly relevant to the present limb-like model, it has been shown that localized tissue heating is strongly correlated to electrode geometry, skin electrical properties, electrode-skin contact conditions, and current/charge density concentration (Gomez-Tames et al., 2016; Khadka and Bikson, 2020). Previous studies have shown that localized current characteristics may substantially affect superficial hotspot formation and temperature elevation during electrical stimulation (Gomez-Tames et al., 2016; Khadka and Bikson, 2020). For example, Gomez-Tames et al. (2016) demonstrated through coupled electrical–thermal modeling that microscopic skin structure and electrode-interface conditions can influence local electric-field concentration and skin temperature elevation during tDCS conditions.

These characteristics are currently not well understood for HD-inspired small electrode stimulation configurations in a peripheral transcutaneous context. As such, it is of interest to explore the relationship between HD montage configuration and active electrode number under controlled geometry conditions. Earlier array electrode studies spanning both transcranial and transcutaneous stimulation contexts have largely focused on electric field distribution and neural activation performance rather than the electrothermal consequences of increasing electrode count (Villamar et al., 2013; Datta et al., 2009; Guler et al., 2016; Jain et al., 2023); notably (Datta et al., 2009; Guler et al., 2016), characterized electric field focality specifically within transcranial HD-tDCS head models, and their findings are referenced here only as general precedent for electrode-subdivision effects on field distribution, not as thermal or electrothermal findings applicable to the present peripheral limb model. To the authors’ knowledge, no prior study has explored the electrothermal behavior of progressive electrode subdivision under fixed electrode geometry conditions in a peripheral transcutaneous surface neurostimulation context.

Specifically, increasing the number of subdivided electrodes may not produce a linear change in tissue heating behavior.

The objective of this study was to develop a computational model to understand how electrode numbers in an HD neurostimulation configuration influences electrothermal behavior during pulsed surface neurostimulation. To address this problem, a coupled electrical–thermal finite-element model was developed to compare tissue heating across different configurations. This includes a conventional single-electrode configuration (i.e., reference configuration) as well as a systematic series of HD-inspired small-electrode grid configurations ranging from 1 to 16 electrodes (grid1 through grid16), in which individual electrode radius and inter-electrode spacing were held constant while electrode count was systematically varied.

The investigated configurations represent progressive increases in the active electrode number and active area within a single stimulation pole. In this way, we explore the influence of the active-region continuity on current-distribution topology and thermal accumulation. This computational approach enables fundamental electrothermal exploration prior to in-human studies, where the effect of electrode count can be isolated from confounding geometric variables.

The primary contribution of this work is a comparative electrothermal analysis characterizing how HD-inspired electrode subdivision governs current-distribution topology and tissue heating across a systematic grid1-through-grid16 progression. It will be demonstrated that stimulation-side electrothermal load declines continuously with increasing electrode count, following an approximate power-law relationship rather than converging toward conventional single-electrode behavior, with a crossover at which stimulation-side load falls below a fixed return-electrode background contribution identified at $N^{*}=8$ –9. Dedicated area-matched and count-matched control comparisons show that electrode count and total active area—intrinsically coupled across the grid1-through-grid16 progression—each contribute to this decline, though these controls were not factorially designed and do not isolate fully independent causal effects. At the opposite, sparse extreme, the grid1 configuration reproduces the well-established current-concentration effect of small isolated electrodes. These results indicate that electrode count and total active area jointly shape current-distribution topology and the resulting tissue heating. The results provide a comparative computational framework informing the design of high-density cluster electrode technologies and their use workflows in future applications.

## 2 Methods

### 2.1 Tissue geometry

A multilayer planar tissue model was constructed in COMSOL to represent the superficial tissue region beneath the stimulation interface. The tissue domain consisted of four stacked horizontal layers: an effective superficial skin layer, dermis, subcutaneous fat, and muscle tissue. The layer thicknesses and lateral dimensions were selected to represent superficial limb regions, such as the forearm, which is a common application site for wearable transcutaneous neurostimulation systems (Liu et al., 2024; RaviChandran et al., 2023).

The effective superficial skin, dermis, fat, and muscle layers were assigned thicknesses of 0.12 mm, 2 mm, 5 mm, and 10 mm, respectively, based on representative values reported in previous bioelectromagnetic and electrothermal modeling studies (Gabriel et al., 1996). All tissue layers were modeled with an area of 150 × 150 ${\text{mm}}^{2}$ to represent a fixed volume. This lateral extent was selected to place the external boundaries sufficiently far from the stimulation region to minimize boundary effects, as further assessed through the domain-size verification described in Section 2.9.

The stratum corneum and epidermis were combined into a single effective superficial skin layer. This simplification was introduced to avoid excessive local mesh refinement requirements associated with direct discretization of the thin stratum corneum, while preserving the through-thickness electrical resistance of the combined superficial skin region. The equivalent electrical conductivity of this layer was calculated using a series-resistance approximation, as described in Section 2.2. Material properties for all tissue layers are summarized in Section 2.2.

**FIGURE 1:**
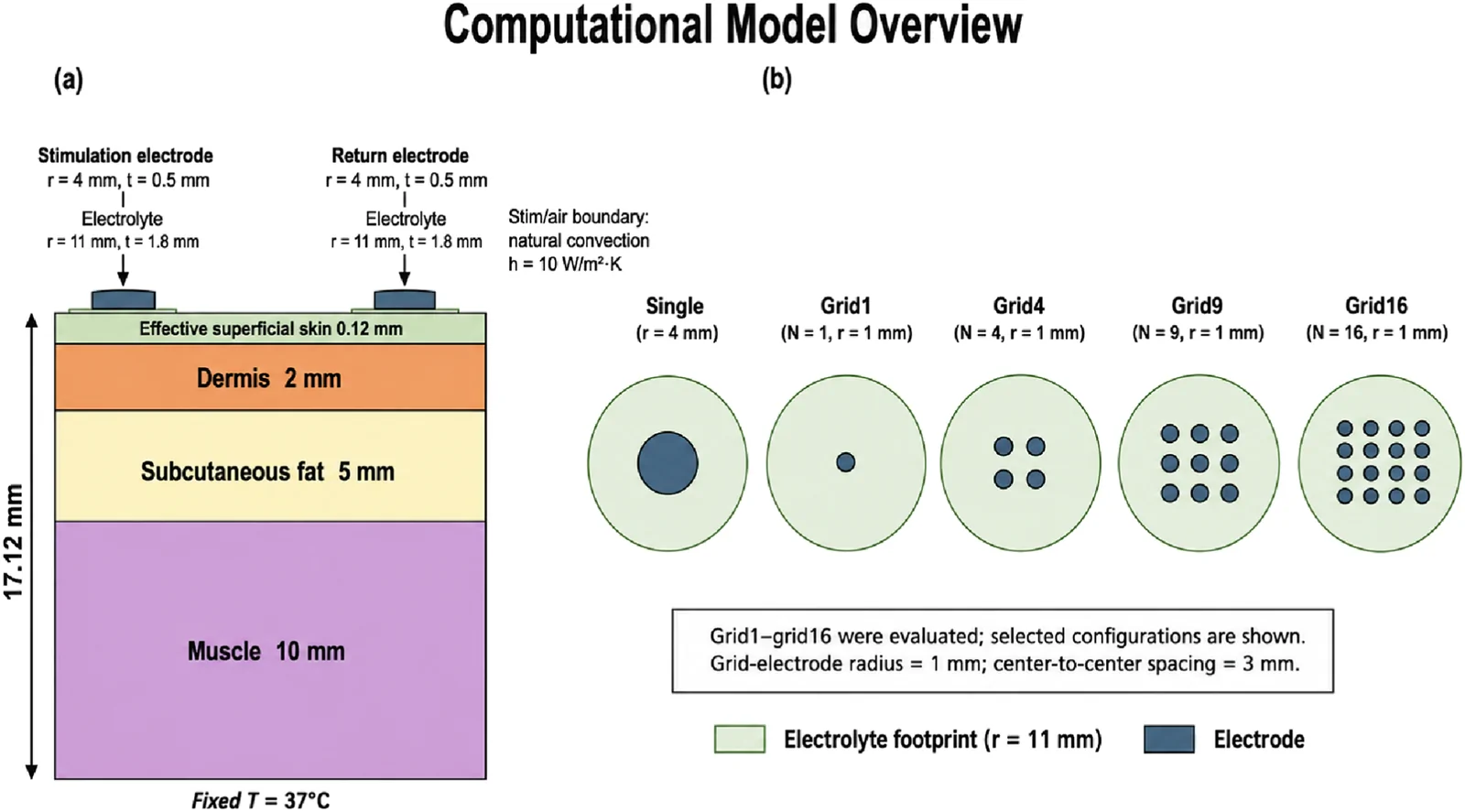
Computational model overview. **(a)** Cross-sectional schematic of the multilayer tissue domain showing layer thicknesses, electrolyte layer dimensions (radius 11 mm, thickness 1.8 mm), and electrode geometry (radius 4 mm, thickness 0.5 mm), with stimulation and return electrode positions (center-to-center separation: 64 mm). Not drawn to scale. **(b)** Top-view schematics of representative stimulation electrode configurations: single electrode (radius 4 mm) and HD-inspired grid1, grid4, grid9, and grid16 configurations (individual electrode radius 1 mm; center-to-center spacing 3 mm), shown over the shared electrolyte layer footprint (radius 11 mm). Grid configurations from grid1 through grid16 were evaluated; selected configurations are shown.

The overall computational domain, tissue layer configuration, and electrode layouts are illustrated in Figure 1.

### 2.2 Material properties

Electrical and thermal material properties were assigned to all tissue and electrode-interface domains using representative values reported in previous bioelectromagnetic and electrothermal modeling studies (Gabriel et al., 1996; Pennes, 1948).

The material properties used in all simulations are summarized in Table 1.

**TABLE 1:** Material parameters assigned to tissue, electrolyte, and electrode domains.

Domain	Density (kg/ $m^{3}$ )	Elec. cond. (S/m)	Thermal cond. (W/m ⋅ K)	Heat capacity (J/kg ⋅ K)	Source
Eff. Superficial skin	1,200	0.0012	0.21	3,600	Calculated; Gabriel et al. (1996); Gomez-Tames et al. (2016); Pennes (1948)
Dermis	1,100	0.30	0.45	3,300	Gabriel et al. (1996); Gomez-Tames et al. (2016); Pennes (1948)
Fat	970	0.020	0.21	2,300	Gabriel et al. (1996); Gomez-Tames et al. (2016); Pennes (1948)
Muscle	1,050	0.30	0.49	3,421	Gabriel et al. (1996); Gomez-Tames et al. (2016); Pennes (1948)
Electrolyte	1,000	0.10	0.60	4,180	Gabriel et al. (1996); Gomez-Tames et al. (2016)
Electrode	10,500	$1.0\times 10^{6}$	150	235	Idealized ${\text{conductor}}^{†}$

†Idealized conductor (not representative of any specific real-world electrode material); see Section 2.2.

^†^The electrode was modeled as an idealized, near-perfect conductor rather than being matched to a specific real-world electrode material. The assigned electrical conductivity ( $1.0\times 10^{6}$ S/m) is approximately six orders of magnitude higher than the highest tissue conductivity in the model (0.30 S/m, dermis/muscle; Table 1), ensuring that the electrode surface behaves as a near-equipotential boundary under the present modeling assumptions regardless of the exact metal identity; the specific choice of density and thermal conductivity values accordingly has negligible influence on the reported electrical and thermal results, which are governed by the tissue-side conduction and heat transport described in Sections 2.4–2.5.

For the effective superficial skin layer, the equivalent electrical conductivity was calculated using a series-resistance approximation to represent the combined stratum corneum and epidermis (Equation 1):

$$\sigma_{\text{eff}}=\frac{d_{\text{SC}}+d_{\text{epi}}}{\frac{d_{\text{SC}}}{\sigma_{\text{SC}}}+\frac{d_{\text{epi}}}{\sigma_{\text{epi}}}}$$ (1)

where $d_{\text{SC}}$ and $d_{\text{epi}}$ represent the thicknesses of the stratum corneum and epidermis, and $\sigma_{\text{SC}}$ and $\sigma_{\text{epi}}$ denote their respective electrical conductivities. Using $d_{\text{SC}}=0.02$ mm, $d_{\text{epi}}=0.10$ mm, $\sigma_{\text{SC}}=0.0002$ S/m, and $\sigma_{\text{epi}}=0.018$ S/m yielded $\sigma_{\text{eff}}\approx 0.0012$ S/m, consistent with previously reported values for superficial skin electrical resistance Gabriel et al., 1996; Gomez-Tames et al., 2016).

### 2.3 Electrode configurations

#### 2.3.1 Single-electrode configuration

A conventional 8 mm single-electrode configuration was used as the baseline/reference configuration for comparison. The stimulation electrode and return (i.e., reference) electrode were modeled as cylindrical metallic conductors with identical geometries, each having a radius of 4 mm and a thickness of 0.5 mm. These dimensions were selected to represent standard electrodes placed with a compact surface stimulation interface in wearable transcutaneous neurostimulation devices.

A cylindrical conductive electrolyte layer was positioned between each electrode and the tissue surface to represent hydrogel-assisted electrode-skin coupling, which is commonly used in transcutaneous stimulation interfaces to avoid direct electrode-skin contact (Malešević et al., 2012). The electrolyte layer had a radius of 11 mm and a thickness of 1.8 mm. The stimulation and return electrodes were positioned on the tissue surface with a center-to-center separation distance of 64 mm, a value within the range commonly employed in transcutaneous surface neurostimulation applications (Maffiuletti, 2010).

#### 2.3.2 Small-electrode grid configurations

Sixteen HD-inspired small-electrode grid configurations were constructed (grid1 through grid16), consisting of 1–16 cylindrical stimulation electrodes arranged in a square-grid pattern. Each small electrode had a radius of 1 mm and a thickness of 0.5 mm, with a fixed center-to-center spacing of 3 mm between adjacent electrodes based on the physical layout of the device design.

The electrode configurations were organized into three spatial frames of increasing size. Grid1 consisted of a single centrally placed electrode. Grid2 through grid4 were constructed by filling positions within a 2×2 grid frame. Grid5 through grid9 were filled within a 3×3 frame, and grid10 through grid16 were filled within a 4×4 frame. Within each frame, electrode positions were filled sequentially in row-major order (left-to-right, top-to-bottom), chosen to reflect a realistic HD electrode fabrication scenario in which a fixed physical contact layout is selectively activated rather than being freshly optimized for each electrode count.

To quantify the sensitivity of this choice, three alternative fill patterns for the grid14 configuration (14 of 16 positions active within the 4×4 frame) were compared under the representative stimulation condition ( I=50 mA, PW =700 μ s, F=10 Hz, t=300 s). Those being, (i) a row pattern (positions 15 and 16 vacant); (ii) two non-adjacent vacancy patterns (e.g., positions 6 and 11 vacant, or positions 6 and 10 vacant); and (iii) an interior vacancy, rather than at the edge or corner. Although the total active electrode area and electrode count are identical across all three patterns, the spatial arrangement of vacancies alters the effective footprint of the active electrode subset to measure the stimulation-side electrothermal behavior (see Section 3.1). The stimulation-side and return-side contributions were evaluated separately for this comparison (Section 3.1).

Current-controlled boundary conditions were applied such that the total stimulation current was held constant across all configurations. Although a pulsed waveform was applied, the electrical field distribution was first solved under stationary conditions for each current amplitude, and the resulting Joule heating source was subsequently scaled by the duty cycle to obtain a duty-cycle-averaged heat source applied continuously in the transient thermal simulation, as described in Sections 2.5.2 and 2.6. For a grid containing N small electrodes, each individual small electrode was assigned an equal current of $I_{\text{total}}/N$ . Consequently, all grid configurations delivered the same total stimulation current as the single-electrode configuration, while the current assigned to each individual small electrode decreased as N increased. This imposed equal-current allocation was used to isolate the influence of active electrode number on electrothermal behavior and was not intended to represent optimized multichannel current steering.

The equal-current allocation reflects an assumed target device architecture in which each small electrode within the grid array is driven by a current-controlled channel. This is consistent with multichannel current-steering hardware architectures used in some HD stimulation systems. Under a common-potential architecture, the N contacts would instead share a single applied potential, and current would redistribute passively across contacts according to local contact impedance, geometry, and tissue properties rather than being held equal. This alternative requires a fundamentally different boundary-condition formulation and so a common-potential comparison was not performed herein. We acknowledge that under a common-potential architecture, current is expected to redistribute non-uniformly across contacts based on local impedance and tissue geometry rather than remaining equal. This affects current-density concentration patterns (particularly in sparse configurations) where locally concentrated current pathways are more sensitive to this boundary-condition. The equal-current assumption used herein is therefore not a general prediction applicable to all possible HD electrode driving strategies (see also Section 4.3).

The return electrode geometry, electrolyte geometry, electrode thickness, and interfacial coupling assumptions were kept identical across all configurations to ensure that observed differences in electrothermal behavior could be attributed to active electrode number.

#### 2.3.3 HD-inspired subdivision design strategy

The electrode grid configurations represent progressive increases in active contact number within a single stimulation pole. This follows an HD-inspired design philosophy in which a conventional stimulation surface is replaced by an array of smaller electrode elements. Individual electrode radius and inter-electrode spacing were held constant across all grid configurations, with electrode count serving as the sole geometric variable. Consequently, the total active electrode area and overall spatial extent of the stimulation array increased with electrode count, while the geometry of each individual electrode element remained unchanged.

The investigated grid1–grid16 configurations therefore differ from conventional fixed-footprint or area-matched electrode comparison approaches, where the total active area or stimulation coverage is held constant. Instead, this primary series reflects a practical wearable device design scenario in which the number of electrode elements is varied while individual electrode geometry and inter-element spacing are constrained by device fabrication and interface requirements. All tissue materials were modeled as homogeneous and isotropic with temperature-independent material properties, to support a controlled comparative analysis across electrode configurations rather than patient-specific prediction. The isotropic muscle assumption in particular is a simplification. For example, skeletal muscle electrical conductivity is known to be direction-dependent, with conductivity along the muscle fiber direction typically several-fold higher than in the transverse direction, and fiber orientation relative to the electrode pair can influence current distribution, depth of current penetration, and stimulation selectivity. This simplification was adopted because the present study’s primary focus is the electrothermal behavior at the superficial skin-electrode interface, where the reported three-regime structure and area-dependent heating patterns are established (Sections 3.1–3.3). Muscle anisotropy is expected to primarily influence deeper current penetration pathways and neural/muscular stimulation selectivity rather than the superficial current-concentration mechanisms that drive the reported ΔT differences among configurations. This remains an assumption that has not been justified through sensitivity analysis in the present study (Section 4.3).

#### 2.3.4 Area-matched control configurations

To directly disentangle electrode count from total active electrode area a dedicated area-matched experiment was constructed. Specifically, the individual electrode radius was increased so that the total active electrode area $\left(N\times \pi r^{2}\right)$ matched that of the single-electrode reference configuration $\left(\pi \times 4^{2}\;{mm}^{2}\approx 50.27\;{mm}^{2}\right)$ , while the electrode count was held to N=4 (AM-Grid4, r=2 mm) and N=9 (AM-Grid9, r=1.333 mm). The center-to-center spacing was increased to 5 mm for AM-Grid4 and 3.667 mm for AM-Grid9. Thus, in both cases the original 1 mm edge-to-edge gap between adjacent electrodes ( spacing=2r+1 mm) was preserved. All other aspects of the model (i.e., return electrode geometry, electrolyte geometry, tissue layer properties, and boundary conditions) were kept identical to the primary grid1–grid16 series. The area-matched configurations were evaluated across the full 128-combination stimulation parameter sweep described in Section 2.7, using the same electrothermal metrics as the primary series. The stimulation-side/return-side/global decomposition described in Section 3.1 was additionally applied to these configurations, together with a complementary, exploratory count-matched comparison. For example, grid4 and grid9 were compared against their area-matched analogue at fixed electrode count using the representative stimulation condition. Results of this control analysis are reported in Section 3.2.

### 2.4 Electrical model

#### 2.4.1 Governing equation

Electrical current transport was modeled using the electroquasistatic approximation, which is appropriate for low-frequency bioelectric stimulation analysis where conductive current transport dominates displacement current effects (Datta et al., 2008; Saturnino et al., 2015). In the present simulations, the stimulation frequencies ranged from 10 to 70 Hz, which are well within the low-frequency regime used for biological tissues (Datta et al., 2008; Saturnino et al., 2015).

The electric potential distribution was defined by the current continuity equation (Equation 2):

$$\nabla \cdot \left(\sigma \nabla V\right)=0$$ (2)

where σ is the electrical conductivity and V is the electric potential. The electric field and current density were derived as (Equation 3):

E=−∇V,J=σE (3)

where E is the electric field vector and J is the current density vector.

#### 2.4.2 Boundary conditions

Current-controlled stimulation was applied in all simulations. A fixed total current was prescribed at the terminal boundary of the stimulation electrode or electrode array, and the return electrode terminal was assigned a ground potential boundary condition (V=0) . For grid configurations, the total applied current was partitioned equally across the N small electrodes by assigning an identical terminal current of $I_{\text{total}}/N$ to each individual electrode, as described in Section 2.3.2.

Electrical insulation was applied to the external lateral and bottom boundaries of the tissue domain to prevent artificial current leakage (Equation 4):

n⋅J=0 (4)

where n is the outward surface normal vector. Ideal electrical continuity was assumed at all internal material interfaces, including electrode-electrolyte and electrolyte-tissue boundaries. Contact impedance and interfacial polarization effects were not explicitly modeled, consistent with the idealized hydrogel-assisted contact assumption described in Section 2.3.1. This idealization and its implications for interpreting the reported results are discussed in Section 4.3. This simplification was adopted to ensure that observed differences in electrothermal behavior across the seventeen electrode configurations could be attributed to active electrode number rather than interfacial variability.

### 2.5 Thermal model

#### 2.5.1 Bioheat equation

Thermal transport was modeled using the Pennes bioheat transfer formulation (Equation 5; Pennes 1948):

$$\rho C_{p}\frac{\partial T}{\partial t}=\nabla \cdot \left(k\nabla T\right)+Q_{\text{bio}}+Q_{\text{ext}}$$ (5)

where ρ is the tissue density, $C_{p}$ is the specific heat capacity, T is temperature, k is the thermal conductivity, $Q_{\text{bio}}$ is the perfusion-mediated bioheat term, and $Q_{\text{ext}}$ is the volumetric heat source generated by electrical energy dissipation.

Tissue perfusion was not included in the present model $\left(Q_{\text{bio}}=0\right)$ . This assumption was adopted to isolate electrode-induced Joule heating from perfusion-mediated thermal effects and to avoid introducing uncertain layer-specific perfusion parameters. As a consequence, the simulated temperature rises represent conservative upper-bound estimates of localized thermal accumulation, as perfusion-mediated cooling is absent. The volumetric Joule heating source $Q_{\text{ext}}$ is described in Section 2.5.2.

#### 2.5.2 J heating source

The volumetric Joule heating source was calculated from the electric field solution (Equation 6):

$$Q_{\text{ext}}=J\cdot E=\sigma |E{|}^{2}$$ (6)

where J is the current density vector, E is the electric field vector, and σ is the electrical conductivity.

For pulsed stimulation, a duty-cycle-averaged Joule heating source was applied in the transient thermal simulation, rather than explicitly resolving each rectangular current pulse in time. Each stimulation cycle consists of a charge-balanced biphasic pulse (two phases, each of duration PW, per cycle), giving an overall duty cycle of 2×PW×F . The time-averaged Joule heating source was computed as (Equation 7):

$$Q_{\text{avg}}=Q_{\text{ext}}\times 2\times PW\times F$$ (7)

where $Q_{\text{ext}}=J\cdot E=\sigma |E{|}^{2}$ is the instantaneous Joule heating density obtained from the stationary electrical solution at the prescribed current amplitude (see equation above), and 2×PW×F is the fraction of each stimulation cycle during which current actually flows. This duty-cycle-averaged approach is appropriate given the thermal time constants of the tissue layers modeled here (on the order of seconds to tens of seconds, Section 2.5.1), which are many orders of magnitude longer than the microsecond-scale pulse widths (100–700 μ s) investigated in this study. The tissue thermal response to a rapid, repeating sequence of short current pulses is expected to closely track the time-averaged delivered power rather than resolve the microsecond-scale on/off structure of individual pulses. This is also consistent with the transient thermal solver settings described in Section 2.6 (adaptive time stepping with a 1 s initial time step), which would not otherwise be capable of resolving individual sub-millisecond pulse events.

#### 2.5.3 Thermal boundary conditions

The top surfaces of the electrodes and the exposed electrolyte region, as well as the bottom boundary of the muscle layer, were assigned a fixed temperature of 37 °C to be consistent with core body temperature and to provide thermal coupling with deeper body tissues (Pennes, 1948). No convective heat-exchange boundary condition was applied to any external surface. The lateral boundaries of the tissue domain were left unassigned and were therefore thermally insulated by COMSOL’s default zero-flux boundary condition. Perfect thermal continuity was assumed at all internal material interfaces, and thermal contact resistance was neglected to maintain consistency with the electrical interface assumptions described in Section 2.4.2.

To verify that this boundary configuration does not introduce a passive baseline temperature drift that could confound the reported stimulation-induced ΔT , a zero-current (Q=0) control simulation was performed for representative configurations (grid1 and grid4), with all other boundary conditions unchanged. The temperature remained at the uniform 37 °C initial condition throughout the simulated duration ( |ΔT|<0.001 °C at all nodes and all time points), confirming that the reported ΔT values reflect stimulation-induced heating only, with no measurable passive drift requiring correction (see also Section 4.3).

### 2.6 Numerical implementation

All simulations were performed in COMSOL Multiphysics 6.3 (COMSOL Inc., Burlington, MA, USA) using the Electric Currents and Bioheat Transfer physics interfaces. Both physics interfaces used quadratic Lagrange shape functions (electric potential and temperature, respectively). A physics-controlled mesh with normal element size was employed, with automatic local refinement applied by the physics-controlled meshing algorithm near the electrode-tissue interface regions where steep gradients in current density and temperature are expected. No additional boundary-layer or manually specified local mesh refinement was applied beyond this default physics-controlled algorithm. For the production (Normal) mesh, this corresponded to 991,491 degrees of freedom for the grid4 configuration and 1,074,768 degrees of freedom for the grid1 configuration (Section 2.9).

The coupled electrothermal problem was solved using a sequential one-way coupling workflow. In the first stage, the electrical conduction problem was solved under stationary conditions for each stimulation current amplitude and electrode configuration, yielding the spatial distributions of electric potential, electric field, and current density. In the second stage, the resulting electric field distribution was used to compute the instantaneous volumetric Joule heating density $Q_{\text{ext}}$ , which was then scaled by the duty cycle (2×PW×F) to obtain the duty-cycle-averaged heat source $Q_{\text{avg}}$ applied continuously in the transient thermal simulation (Section 2.5.2). No temperature-dependent feedback on electrical conductivity was included, consistent with the temperature-independent material property assumption described in Section 2.2.

The transient thermal problem was solved over a stimulation duration of 300 s using an implicit backward differentiation formula (BDF) time-dependent solver with adaptive time stepping. The relative tolerance was set to 0.01, with an initial time step of 1 s and automatic maximum time-step control. The maximum and minimum BDF orders were set to 2 and 1, respectively. These solver settings were selected to balance computational efficiency with numerical accuracy, and their adequacy was confirmed through the mesh convergence and numerical robustness evaluation described in Section 2.9.

### 2.7 Stimulation parameter sweep

A parametric sweep was performed to systematically evaluate how stimulation current amplitude, pulse width, and stimulation frequency influenced electrothermal behavior across the seventeen electrode configurations. Eight current amplitude levels (10, 20, 30, 40, 50, 60, 70, and 80 mA), four pulse-width levels (100, 300, 500, and 700 μ s), and four frequency levels (10, 30, 50, and 70 Hz) were sampled, yielding 8×4×4=128 parameter combinations per electrode configuration. These ranges were selected to encompass typical clinical TENS/NMES operating conditions together with an extended upper range included specifically to probe electrothermal transition boundaries. Typical reported TENS/NMES operating conditions are generally on the order of I=10 –50 mA, PW =100 –400 μ s, and F=10 –50 Hz Maffiuletti (2010). The upper portion of the investigated sweep (particularly I>50 mA combined with PW >400 μ s and F>50 Hz) should therefore be interpreted as stress-test conditions probing the electrothermal parameter space beyond typical clinical operation. This was deliberately included to identify electrothermal transition regions rather than to reproduce any specific clinical protocol. The thermal transition results should accordingly be interpreted with this in mind, and are not intended to broadly represent typical clinical use.

The parameter sweep was conducted for each electrode configuration under otherwise identical conditions. For each parameter combination, tissue geometry, material properties, return-electrode geometry, electrolyte dimensions, and thermal boundary conditions were held constant. This design ensured that the observed differences in current density distribution and temperature rise across parameter combinations were relative to stimulation parameters and active electrode number, rather than to confounding geometric or interface variables.

To place the investigated parameter range in dosimetric context, Table 2 reports configuration-level dosimetry at the representative stimulation condition ( $I_{\text{total}}=50$ mA, PW =700 μ s, F=10 Hz). Since per-contact current decreases as $I_{\text{total}}/N$ while individual electrode area is fixed at $\pi r^{2}$ for the grid configurations, the nominal per-contact current density and charge density differ across configurations. The contact current density is calculated as terminal current divided by individual electrode area, prior to any spreading through the hydrogel and tissue. For example, a grid1 should exhibit a nominal charge density approximately 16 × that of the single-electrode reference at this condition, whereas grid16 should be dosimetrically identical to single. At the upper end of the investigated sweep ( $I_{\text{total}}=80$ mA, PW =700 μ s, F=70 Hz), grid1 applies a nominal charge density of approximately 1783 μ C/ ${cm}^{2}$ per pulse. This is well above safety ranges typically used in transcutaneous stimulation. The duty cycle ( 2×PW×F , Section 2.5.2) ranged from 0.2% (PW =100 μ s, F=10 Hz) to 9.8% (PW =700 μ s, F=70 Hz) across the sweep and is independent of electrode configuration.

**TABLE 2:** Configuration-level dosimetry at the representative stimulation condition ( $I_{\text{total}}=50$ mA, PW =700 μ s, F=10 Hz; duty cycle =1.4% for all configurations, 2×PW×F , Section 2.5.2). Nominal current density and charge density are computed as terminal current divided by individual electrode geometric area, prior to spreading through the hydrogel/tissue, and therefore represent an upper-bound dosimetric estimate at the electrode terminal rather than the simulated in-tissue current density reported in Section 3.1 (e.g., $J_{\mathrm{p99}}$ ).

Config.	N	Total active area $\left({\text{mm}}^{2}\right)$	Array footprint (mm)	Current per contact (mA)	Nominal J per contact (A/ $m^{2}$ )	Charge per pulse ( μ C)	Charge density per pulse ( μ C/ ${cm}^{2}$ )
Single	1	50.27	8	50.00	994.7	35.00	69.6
Grid1	1	3.14	2	50.00	15,915.5	35.00	1,114.1
Grid4	4	12.57	5	12.50	3,978.9	8.75	278.5
Grid9	9	28.27	8	5.56	1768.4	3.89	123.8
Grid16	16	50.27	11	3.13	994.7	2.19	69.6

### 2.8 Electrothermal metrics

Electrothermal response was characterized using two complementary metrics: temperature rise and current density magnitude. Temperature elevation was quantified relative to the initial baseline tissue temperature (Equation 8):

$$\Delta T=T\left(t\right)-T_{0}$$ (8)

where $T_{0}$ is the initial tissue temperature prior to stimulation onset. Maximum temperature rise and hotspot location within each tissue layer were recorded for each stimulation parameter combination and electrode configuration.

The initial tissue temperature field was set to a uniform $T_{0}=37$ °C throughout the domain at t=0 , consistent with the fixed 37 °C thermal boundary conditions applied at all external surfaces, with no convective heat-exchange boundary applied anywhere in the model (Section 2.5.3). Because these boundary conditions are uniform, no pre-stimulation equilibration step was required. This was confirmed by a zero-current (Q=0) control simulation, which showed no measurable passive baseline drift ( |ΔT|<0.001 °C throughout the simulated duration; Section 2.5.3). Absolute maximum temperature $\left(T=T_{0}+\Delta T\right)$ is reported alongside ΔT at key representative and extreme-condition data points in this manuscript to avoid ambiguity (Table 9; Section 3.5).

Current density and temperature rise are evaluated primarily within an explicitly defined stimulation-side region of interest (20 mm radius centered on the stimulation electrode array centroid). This choice was adopted because the fixed return electrode—whose geometry and total current are identical across all seventeen configurations—makes a constant, non-negligible contribution to any unrestricted domain-wide (global) evaluation of current density and temperature rise. Corresponding return-side and global values are reported (Table 6) for completeness, but are not used as the primary basis for the comparative regime analysis reported in Section 3.3 onward.

Two temperature-rise reference thresholds were defined for characterizing electrothermal transition regions, and were not intended to represent clinical tissue injury criteria. A moderate threshold of ΔT=3 °C was selected as a conservative comfort-oriented boundary, motivated by reported changes in cutaneous thermal perception and thermal comfort during skin heating (Mekjavic et al., 2021). A higher threshold of ΔT=8 °C was defined as a reference level motivated by the IEC 60601–1 applied-part surface temperature limit of 41 °C. This limit is conventionally referenced against a resting skin-surface temperature of approximately 33 °C for limb regions (Lee et al., 2019), whereas the present model’s initial and boundary temperature is 37 °C (Section 2.5.3). Under the 37 °C baseline modeled here, the equivalent margin to the 41 °C absolute limit corresponds to ΔT=4 °C. The ΔT=8 °C threshold should therefore be understood as a conservative, round-number reference retained for comparability with the wider stimulation-safety literature which commonly assumes a lower resting skin baseline than 37 °C. Furthermore, the IEC 60601–1 applied-part limit refers to the temperature of the device surface in contact with the patient, not to subsurface tissue temperature. Thus, the ΔT values reported herein describe tissue-layer temperature rise rather than applied-part surface temperature, and this distinction should be kept in mind when relating the reported thresholds to the IEC limit. Hotspot locations, distinguishing electrode material, hydrogel, and superficial skin tissue, are reported alongside the absolute maximum temperature already noted above (Section 3). These two thresholds together defined three parameter-space regions— ΔT<3 °C, 3≤ΔT<8 °C, and ΔT≥8 °C—applied consistently across all electrode configurations and stimulation parameter combinations to enable direct comparative electrothermal analysis.

Current density magnitude was evaluated in conjunction with temperature rise since the electrode-tissue interface directly governs the spatial distribution of Joule heat generation. Because the model uses ideal metallic electrodes with sharp geometric boundaries, the raw nodal-maximum current density $\left(J_{\text{max}}\right)$ near an electrode edge is subject to a classic edge singularity—corroborated by an analytical disc-electrode benchmark rather than being an artifact confined to the finite-element discretization (Section 2.9). To obtain a more robust and reproducible comparative metric, the 99th-percentile current density $\left(J_{\mathrm{p99}}\right)$ is reported as the primary current-density metric throughout this study, alongside the raw $J_{\text{max}}$ for reference.

Consistent with the stimulation-side/return-side/global decomposition applied to ΔT above, $J_{\mathrm{p99}}$ and $J_{\text{max}}$ are each reported over three explicitly defined evaluation domains. A stimulation-side region of interest (20 mm radius centered on the stimulation electrode array centroid), a return-side region of interest (20 mm radius centered on the fixed return electrode), and the global effective superficial skin layer encompassing the full model domain. Every reported current-density value in this manuscript is labeled with its corresponding evaluation domain to avoid the ambiguity that can otherwise arise (Section 4.3).

Because the raw nodal $J_{\mathrm{p99}}$ is computed directly over unstructured mesh nodes, its value is weighted by local mesh-node density rather than by physical tissue area. A supplementary area-weighted $J_{\mathrm{p99}}$ was therefore also computed by resampling the current-density field onto a uniform spatial grid (0.25 mm resolution) prior to percentile calculation, to test the sensitivity of this metric to the underlying node-density weighting. Mesh-convergence analysis (Section 2.9) found that neither the nodal nor the area-weighted $J_{\mathrm{p99}}$ fully converged within the range of mesh refinement levels tested. Both should therefore be interpreted as descriptive, comparative metrics evaluated on a fixed, uniformly-applied production mesh, rather than as mesh-independent absolute values.

$J_{\mathrm{p99}}$ and spatial current density distributions were analyzed alongside the temperature change to establish the link between active electrode number, current-distribution topology, and the resulting electrothermal response across the investigated configurations.

### 2.9 Numerical validation

Numerical verification was performed to assess mesh independence, domain-size independence, and physical consistency of the coupled electrothermal simulation framework prior to conducting the full parametric sweep.

Mesh convergence analysis was conducted by refining the computational mesh while monitoring the peak current density and maximum temperature rise near the electrode-tissue interface using the grid4 configuration under representative stimulation conditions ( I=50 mA, PW =700 μ s, F=10 Hz). Five mesh refinement levels were evaluated: Coarser, Normal, Fine, Finer, and Extra Fine. Successive refinement levels were compared until further refinement produced changes below 2%. The Normal mesh level satisfied this criterion, with the most refined level (Extra Fine) differing from Normal by only 0.62% in maximum temperature rise. The final mesh configuration, as described in Section 2.6, was selected on this basis. Mesh statistics across all refinement levels are summarized in Table 3.

**TABLE 3:** Mesh convergence statistics across refinement levels for the grid4 configuration under representative stimulation conditions ( I=50 mA, PW =700 μ s, F=10 Hz).

Mesh level	Elements	DOF	Min. Element quality	Peak J (A/ $m^{2}$ )	Max ΔT (°C)
Coarser	161,040	250,278	0.007266	769.41	2.760
Normal	672,084	991,491	0.007623	751.31	2.759
Fine	797,590	1,158,867	0.01051	758.88	2.749
Finer	1,492,243	2,152,339	0.01706	757.18	2.740
Extra fine	13,109,500	18,558,111	0.05628	762.71	2.742

The minimum element-quality statistic reported in Table 3—COMSOL’s built-in dimensionless measure (0–1 scale) of the shape regularity of the single worst element anywhere in the mesh—improved monotonically with mesh refinement (0.007266, 0.007623, 0.01051, 0.01706, and 0.05628 for Coarser through Extra Fine, respectively), confirming that progressive refinement systematically improved worst-case element shape quality as expected.

By contrast, the peak current density reported in Table 3 does not vary monotonically with mesh refinement: Normal (751.31 A/ $m^{2}$ ) is the lowest of the five values, below both the Coarser (769.41 A/ $m^{2}$ ) and the three finer (758.88, 757.18, and 762.71 A/ $m^{2}$ ) levels. All five values clustered within a 2.4% band rather than approaching a single limit from one side. This behavior is consistent with the raw nodal-maximum current density’s sensitivity to exact sampling location (Section 3.1). Since the electrode-tissue interface is modeled as an ideal, sharp-edged metallic disc, the current density is expected analytically to diverge at the electrode edge. This expectation is corroborated by the closed-form disc-electrode benchmark presented later in this section. Refining the mesh changes the precise position of the nearest surface node to this singular edge in a manner that is not itself systematically tied to element count, so repeatedly resampling a quantity arbitrarily close to a mathematical singularity is expected to produce a non-monotonic, effectively noise-like sequence rather than a one-sided convergence trend. This reflects a property of the sampled quantity itself rather than evidence of an unconverged or unreliable mesh and stands in direct contrast to the monotonic improvement observed for the mesh-quality statistic above. By contrast, maximum temperature rise—Joule heating over the full 300 s stimulation (Section 2.5.1)—is a smooth, well-posed function of the current-density field and is not expected to inherit this point-sampling sensitivity. Consistent with this expectation, ΔT varied only slightly across all five refinement levels (2.760, 2.759, 2.749, 2.740, and 2.742 °C for Coarser through Extra Fine, respectively), decreasing monotonically (2%) through Finer refinement. There was a negligible (0.07%) uptick at the Extra Fine refinement. The Normal mesh was retained as the primary mesh on this basis: (i) the governing comparative metric, ΔT , is converged to within 0.62% of the most refined level tested (Extra Fine), well inside the stated tolerance; (ii) the non-monotonic behavior of the raw peak current density reflects the sampling sensitivity of a singular quantity rather than a defect in the mesh itself, and is precisely the reason $J_{\mathrm{p99}}$ rather than raw $J_{\text{max}}$ is adopted as the primary current-density metric throughout this study (Section 3.1); and (iii) the Normal mesh (672,084 elements) offers substantially lower computational cost than the finer levels tested. For example, it has 20 × fewer elements than Extra Fine (13,109,500 elements). We note that the Peak J values reported here are evaluated within the same explicitly defined stimulation-side region of interest used throughout the rest of this manuscript (Section 2.8).

The mesh-convergence analysis above was performed using the grid4 configuration, which is representative of the transition-regime configurations but not the most demanding case for mesh resolution. Because grid1 combines the smallest active electrode area and the steepest current-density gradients among the investigated configurations (Section 3.1), a second, independent mesh-convergence study was performed for grid1 under a high-current stress-test condition ( I=80 mA, PW =700 μ s, F=10 Hz) to confirm that the production mesh remains adequate for this worst-case configuration. Results are summarized in Table 4.

**TABLE 4:** Mesh convergence statistics across refinement levels for the grid1 configuration under a high-current stress-test condition ( I=80 mA, PW =700 μ s, F=10 Hz).

Mesh level	Elements	DOF	Min. Element quality	Peak J (A/m^2^)	Max ΔT (°C)
Coarser	157,267	245,282	0.006613	2,604.02	25.712
Normal	735,200	1,074,768	0.01102	2,445.35	27.031
Fine	803,350	1,166,265	0.00976	2,413.76	27.185
Finer	1,483,861	2,140,693	0.01736	2,389.00	27.195
Extra fine	13,084,962	18,524,160	0.05689	2,402.10	27.139

Two features of Table 4 warrant comment. First, the Coarser-to-Normal step shows a comparatively large change in peak current density ( − 6.09%) and maximum temperature rise (5.13%). This is consistent with the Coarser preset being insufficient to resolve the steep current-density gradient around grid1’s small (1 mm radius) electrode edge, and is itself evidence that mesh resolution matters where grid1’s geometry is most demanding. From Normal onward, both metrics are well converged: ΔT varies by no more than 0.57% between any two successive levels (Normal to Extra Fine: 0.40%), and peak J declines essentially monotonically through Finer (Normal to Finer: − 2.30% total) (Table 3). This difference is consistent with the two studies probing different aspects of the same underlying electrode-edge singularity. For example, at grid1’s higher current density and correspondingly sharper local gradient, successive mesh refinements resolve the near-edge field with less positional noise than at grid4’s comparatively more moderate gradient (Section 3.1). Second, the minimum element-quality statistic is not perfectly monotonic here (0.006613, 0.01102, 0.00976, 0.01736, and 0.05689 for Coarser through Extra Fine). Specifically, Fine shows a small dip relative to Normal ( − 11%). Given that this statistic tracks the single worst-shaped element anywhere in the domain (see discussion above), a modest, isolated dip of this kind is consistent with ordinary run-to-run variability in physics-controlled mesh generation. As with the grid4 study above, the Peak J values reported here are evaluated within the same explicitly defined stimulation-side region of interest used throughout the rest of this manuscript (Section 2.8). Together with the grid4 study above, this confirms that the Normal production mesh remains adequate even for the most current-concentrated configuration investigated in this study.

Because $J_{\mathrm{p99}}$ (Section 2.8) rather than raw $J_{\text{max}}$ is adopted as the primary current-density metric throughout this study, a dedicated mesh-convergence check was additionally performed for $J_{\mathrm{p99}}$ . Results are summarized in Table 5.

**TABLE 5:** $J_{\mathrm{p99}}$ (stimulation-side region of interest) across mesh refinement levels for grid4 ( I=50 mA) and grid1 ( I=80 mA), nodal and area-weighted formulations, expressed as percent deviation from the Extra Fine reference level.

Mesh level	grid4 nodal	grid4 area-wt.	grid1 nodal	grid1 area-wt.
Coarser	+29.7%	+17.4%	+43.5%	+36.5%
Normal	+21.6%	+22.6%	+26.5%	+32.3%
Fine	+14.0%	+6.2%	+23.8%	+8.7%
Finer	+7.0%	− 0.1%	+9.1%	− 4.5%
Extra fine	0.0% (ref.)	0.0% (ref.)	0.0% (ref.)	0.0% (ref.)

Unlike ΔT , $J_{\mathrm{p99}}$ did not converge to within the 2% criterion adopted elsewhere in this study at the Normal production mesh, for either the nodal or area-weighted formulation, in either configuration tested. For example, grid1 in particular continued to show a systematic decline even at the finest mesh level tested. We interpret this as a further manifestation of the same electrode-edge current-density singularity discussed above (Table 3; Table 4) rather than a numerical implementation error. Accordingly, $J_{\mathrm{p99}}$ values reported throughout this study should be interpreted as descriptive, comparative metrics evaluated consistently on the fixed Normal production mesh across all configurations, rather than as mesh-independent absolute current-density values. This does not affect the primary comparative metric used for cross-configuration conclusions in this study, ΔT , which was confirmed to converge at the Normal mesh (Tables 5, 6). The area-weighted formulation showed comparatively better convergence behavior at the finer mesh levels for grid4 (Finer-to-Extra-Fine change of − 0.1% versus 7.0% for the nodal formulation), providing some support for area weighting as an improved comparative metric (Section 4.3).

**TABLE 6:** Stimulation-side versus return-electrode-side current density within the epidermis layer, decomposed using a 20 mm-radius region around each electrode’s center ( I=50 mA, PW =700 μ s, F=10 Hz). The return electrode’s contribution is approximately constant across configurations, reflecting its fixed geometry and current; from approximately grid8 onward, this fixed contribution exceeds the stimulation array’s own (declining) current density.

Configuration	Stim-side $J_{\text{max}}$ (A/m^2^)	Stim-side $J_{\mathrm{p99}}$ (A/m^2^)	Return-side $J_{\text{max}}$ (A/m^2^)	Return-side $J_{\mathrm{p99}}$ (A/m^2^)
Grid1	1,528.3	859.2	521.2	476.2
Grid4	751.3	640.7	521.2	476.3
Grid7	535.3	456.1	521.2	474.8
Grid8	501.4	442.9	521.2	474.8
Grid9	466.4	419.4	521.2	474.9
Grid16	355.8	299.7	521.2	474.7

Domain-size verification was performed to assess potential boundary effects arising from the selected lateral tissue dimensions of 150 × 150 ${\text{mm}}^{2}$ . Thus, an enlarged computational domain of 250 × 250 ${\text{mm}}^{2}$ was constructed under otherwise identical conditions and the resulting peak current density and maximum temperature rise near the stimulation region were compared against the baseline domain. The observed differences in both metrics were less than 1%, supporting the use of the 150 × 150 ${\text{mm}}^{2}$ domain size for the comparative analysis. This verification addressed lateral extent only. Sensitivity of the reported results to tissue depth (e.g., the 10 mm muscle layer and its electrically insulating fixed-temperature bottom boundary, Section 2.5.3) was not tested and is discussed as an open assumption in Section 4.3.

Physical consistency of the coupled simulation framework was verified by confirming that maximum temperature rise increased with stimulation current amplitude, pulse width, and frequency across all electrode configurations, and that thermal hotspots were spatially co-located with regions of elevated current density near the electrode-tissue interface. These behaviors are consistent with the expected electrothermal coupling between Joule heating and current density distribution, and support the numerical stability and physical validity of the comparative modeling framework.

As an additional analytical benchmark of the electrical sub-model, the surface current-density distribution beneath an idealized disc electrode was compared against the classical closed-form solution for a disc of radius a held at constant potential and carrying total current I into a homogeneous, semi-infinite conducting medium (Equation 9; Newman 1966):

$$J\left(r\right)=\frac{I}{2\pi a\sqrt{a^{2}-r^{2}}},\;0\leq r<a,$$ (9)

which satisfies $\int_{0}^{a}J\left(r\right)\;2\pi r\;dr=I$ by construction and predicts a current density at the disc center of exactly half the nominal terminal average $\left(J\left(0\right)=0.5\times I/\left(\pi a^{2}\right)\right)$ , and diverging as ${\left(a-r\right)}^{-1/2}$ toward the electrode edge. For the single-electrode configuration ( a=4 mm, I=50 mA), this analytical solution predicts J(0)=497.4 A/ $m^{2}$ against a nominal terminal average of 994.7 A/m^2^ (Table 2). For the grid1 configuration ( a=1 mm, I=50 mA), the corresponding analytical values are J(0)=7957.7 A/m^2^ against an average of 15,915.5 A/m^2^. This closed-form solution assumes a homogeneous semi-infinite medium, which differs from the present layered, finite-domain, electrolyte-mediated geometry (Section 2.1), and is therefore not expected to quantitatively reproduce the simulated in-tissue current density at a specific sampling depth (e.g., $J_{\mathrm{p99}}$ , Section 3.1). However, the qualitative edge-divergence behavior it predicts for any equipotential disc electrode is a generic mathematical consequence of the disc boundary condition itself, independent of the specific medium properties beneath the electrode. This analytical result directly corroborates the classification of the simulated $J_{\text{max}}$ edge singularity (Section 3.1) as a genuine feature of the electrode geometry rather than an artifact confined to the finite-element discretization. This comparison is offered as a qualitative, order-of-magnitude benchmark of the electrical sub-model against an established analytical result.

## 3 Results

### 3.1 Current density distribution

The maximum current density increased approximately linearly with stimulation current amplitude across all tissue layers and electrode configurations, confirming stable current-controlled electrical behavior consistent with the electroquasistatic model formulation described in Section 2.4 (Datta et al., 2008; Khadka and Bikson, 2020).

In the superficial skin layer, the current-density comparisons are reported using 99th-percentile current density $\left(J_{\mathrm{p99}}\right)$ rather than the raw nodal maximum $\left(J_{\text{max}}\right)$ , due to its relation to sampling location and mesh resolution, and it does not represent a physically stable quantity (Section 4.3). Moreover, $J_{\mathrm{p99}}$ is evaluated within an explicitly defined stimulation-side region of interest away from the return-electrode contribution discussed below (Section 3.1). At I=50 mA, the stimulation-side $J_{\mathrm{p99}}$ was 442.4 A/m^2^ for the single electrode, compared with 859.5 A/m^2^ for grid1 (1.94 × single). For examples, this is markedly smaller than in the raw nodal maxima for single (520.3 A/m^2^) versus 1,528.3 A/m^2^ for grid1.

To determine whether the stim-side $J_{\mathrm{p99}}$ values adequately capture the stimulation array’s behavior, current-density values within the epidermis layer were additionally decomposed into a stimulation-side region and a return-electrode-side region (20 mm radius around each electrode’s center) for all seventeen configurations. The return electrode was found to make a constant contribution to the epidermis-layer current density, independent of the stimulation-side configuration ( $J_{\text{max}}\approx 521$ A/m^2^; $J_{\mathrm{p99}}\approx 475$ –476 A/m^2^) across all seventeen configurations (Table 6). For the sparse configurations (grid1–grid7), the stimulation side is larger (e.g., grid1 stim-side $J_{\text{max}}=1528.3$ A/m^2^ compared to a return-side contribution of 521.2 A/m^2^), and a hotspot located at the stimulation side. However, because the stimulation array’s current density decreases monotonically with increasing N (Section 3.1), the stimulation side’s contribution falls below the fixed return-electrode contribution from grid8 onward (e.g., stim-side $J_{\text{max}}=501.4$ A/m^2^ for grid8, versus 521.2 A/m^2^ for the return electrode). For these denser configurations, the current-density and temperature maxima are closer to the return-electrode location rather than remaining on the stimulation side. This does not alter the reported ΔT -based regime structure (Section 3.3), which is evaluated specifically within the stimulation-side region of interest, but it should be considered when interpreting stimulation-array behavior specifically for N≥8 –9 (Section 4.3).

**FIGURE 2:**
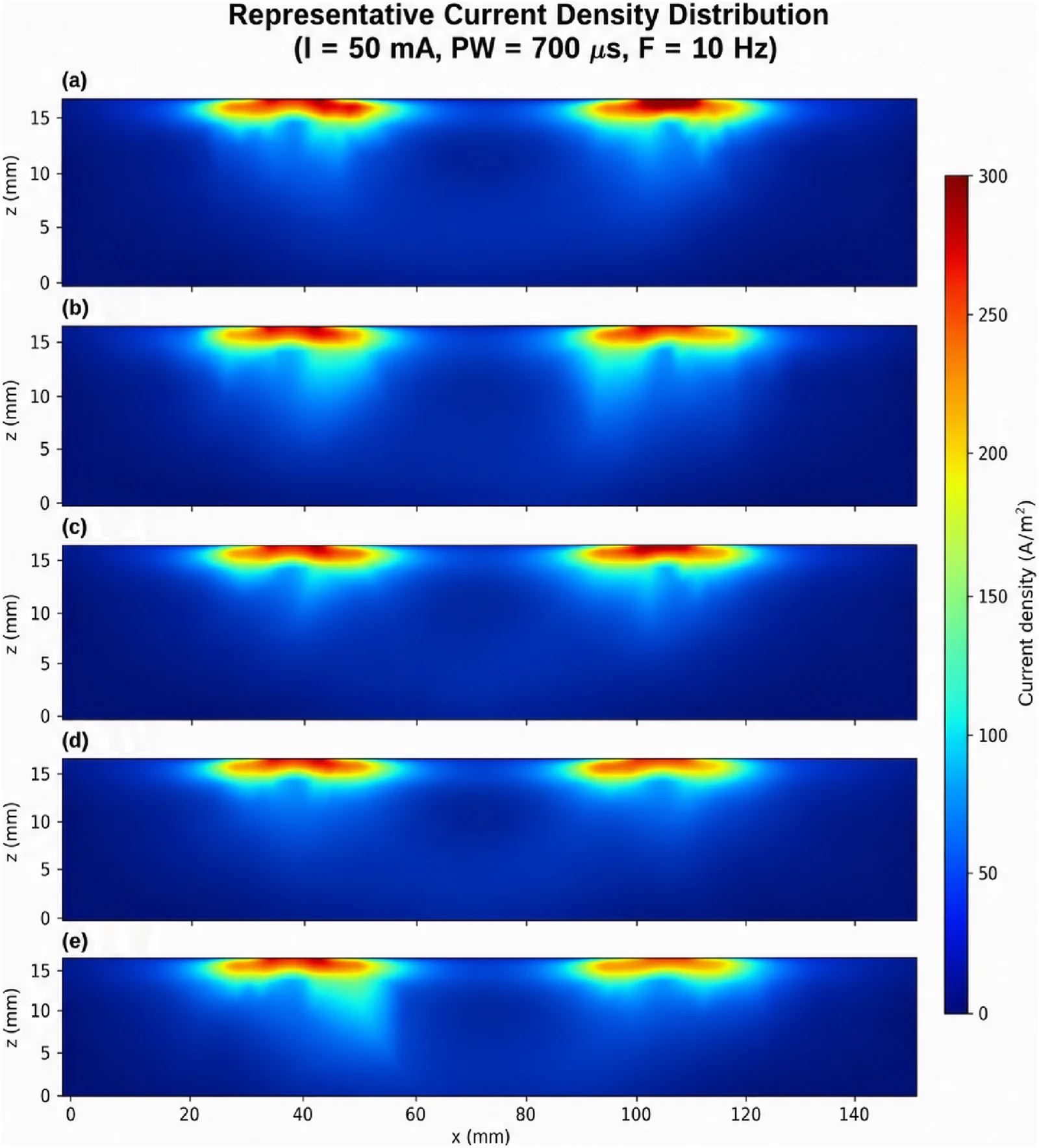
Representative current-density maps for all electrode configurations at matched stimulation conditions ( I=50 mA, PW =700 μ s, F=10 Hz). Cross-sectional view along the central longitudinal axis of the stimulation electrode array, restricted to the tissue domain (excluding the electrode and hydrogel/electrolyte layers above the tissue surface). **(a)** Grid1, **(b)** Single, **(c)** Grid4, **(d)** Grid9, **(e)** Grid16. All panels share a common manual color-table range (0–300 A/m^2^). This was chosen for direct visual comparability across all five panels rather than to match the stimulation-side $J_{\mathrm{p99}}$ scale reported in Section 3.1 and Table 9. Consequently, four of the five configurations—grid1, grid4, single, and grid9, in decreasing order of severity—saturate at the top of the color scale near the electrode edge, while grid16 ( $J_{\mathrm{p99}}=299.7$ A/m^2^) falls within the displayed range. The underlying spatial current-density gradient within the tissue remains visible and qualitatively comparable across all five panels despite this saturation. However, the relative peak intensities among the four saturating configurations cannot be visually distinguished at this color-table range; see Figure 3 and Table 9 for the corresponding quantitative comparison.

**FIGURE 3:**
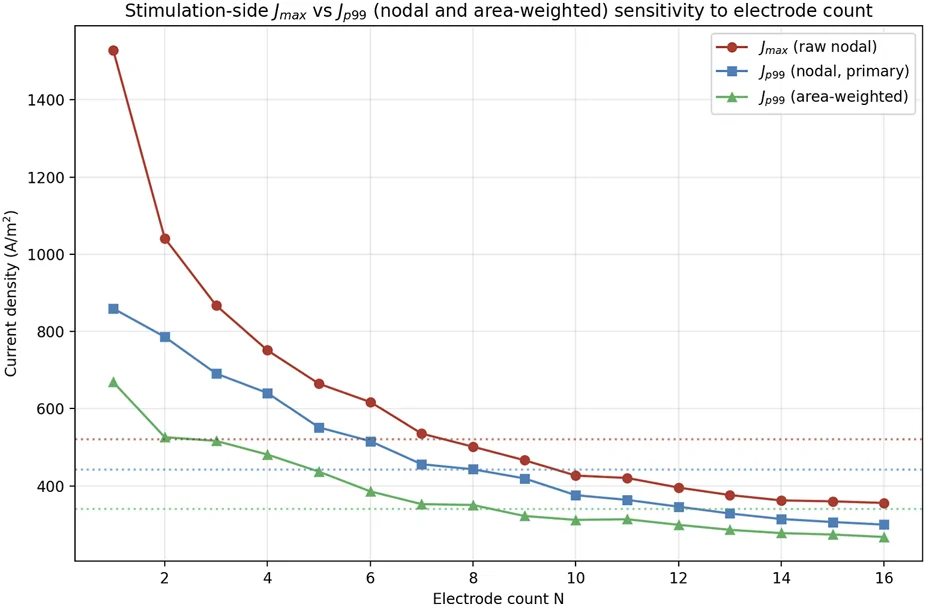
Sensitivity of the raw nodal-maximum current density $\left(J_{\text{max}}\right)$ versus the 99th-percentile current density ( $J_{\mathrm{p99}}$ , both nodal and area-weighted formulations) to electrode count N , stimulation-side region of interest ( I=50 mA, PW =700 μ s, F=10 Hz). Dotted lines indicate the single-electrode reference values for each metric. Stimulation-side $J_{\mathrm{p99}}$ declines continuously with N under both formulations, following a comparably strong approximate power-law relationship in each case (nodal: 1.94 × single at grid1 to 0.68 × single at grid16; area-weighted: 1.96 × to 0.79 × ; Section 3.1); $J_{\text{max}}$ shows a substantially larger apparent range (2.94 × single at grid1), reflecting the mesh- and location-sensitivity of the raw nodal maximum near electrode-edge singularities rather than a proportionally larger underlying physical effect.

The maximum current density across the tissue domain was found to occur within the effective superficial skin layer for all seventeen electrode configurations investigated (Figure 2). Thus, the primary current-density analysis in this study (Section 3.1; Figure 3) is accordingly conducted at this depth. However, to assess whether the stimulation-side or return-side decomposition at the superficial layer reveals comparable footprint-related effects at depth, an equivalent analysis was additionally performed for the dermis layer (thickness 2 mm; Section 2.1).

In the dermis, the stimulation-side $J_{\mathrm{p99}}$ showed a similar declining trend with electrode count (414.7 A/m^2^ for grid1 versus 301.6 A/m^2^ for single, 1.37 × ), but with a notable, reproducible deviation from monotonicity at grid10. In this case, grid10 showed an elevated value (330.0 A/m^2^, 1.09 × single), whereas at grid9 was equivalent to the single electrode (300.3 A/m^2^). The values were then lower in grid11–grid16. The elevated grid10 value does not appear attributable to the fixed return-electrode as it shows consistency across all configurations (dermis return-side $J_{\mathrm{p99}}\approx 303$ –305 A/m^2^), and coincides with the transition from the 3×3 to 4×4 base-grid footprint (Section 2.3.2). In the corresponding superficial-layer $J_{\mathrm{p99}}$ and ΔT trends show no comparable discontinuity. This suggests that current density at depth can be more sensitive to the spatial footprint of the active electrode subset than the superficial layer. This footprint-sensitivity does not measurably propagate into the superficial-layer or ΔT -based comparisons that form the primary basis for the regime structure reported in Section 3.3.

For a direct test of footprint sensitivity within a fixed electrode count, three alternative fill patterns for the grid14 configuration were compared under the representative stimulation condition. Stimulation-side current density showed only modest sensitivity to fill pattern (grid14: 362.38 A/m^2^; vacancy pattern 1 [positions 6, 11]: 353.19 A/m^2^, −2.5% ; vacancy pattern 2 [positions 6, 10]: 347.57 A/m^2^, −4.1% ). This is consistent with $J_{\mathrm{p99}}$ being a percentile-based statistic aggregated over all active electrode edges, and therefore comparatively insensitive to permutations in the vacant positions. In contrast, the stimulation-side ΔT showed a substantially larger, non-negligible sensitivity to fill pattern (grid14: 0.8674 °C; vacancy pattern 1: 0.7352 °C, −15.2% ; vacancy pattern 2: 0.7524 °C, −13.3% ), indicating that the spatial arrangement of active electrode positions exerts a measurable influence on the 300 s integrated thermal response. However, it had comparatively little effect on the instantaneous current-density field.

### 3.2 Area-matched control analysis

To test whether the elevated heating observed in sparse grid configurations reflects electrode count or is instead attributable to the confounded reduction in total active electrode area (Section 2.3.4), the area-matched control series (AM-Grid4, N=4 , r=2 mm; AM-Grid9, N=9 , r=1.333 mm) was evaluated under the representative stimulation condition ( I=50 mA, PW =700 μ s, F=10 Hz, t=300 s) using the stimulation-side/return-side/global decomposition described in Section 3.1.

**FIGURE 4:**
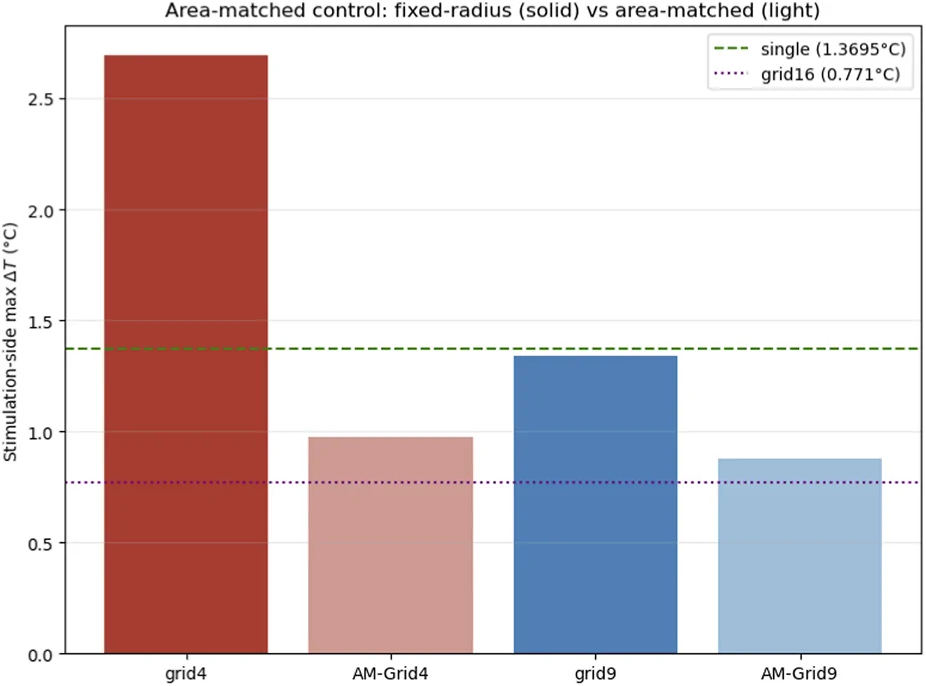
Area-matched control analysis. Stimulation-side maximum temperature rise (ΔT) at N=4 and N=9 under fixed-radius ( r=1 mm, original grid N series) versus area-matched (50.27 ${\text{mm}}^{2}$ ) conditions ( I=50 mA, PW =700 μ s, F=10 Hz, t=300 s). The dashed line indicates the single-electrode stimulation-side reference ΔT (1.3695 °C).

Under the representative condition, the stimulation-side ΔT for AM-Grid4 and AM-Grid9 was 0.9753 °C and 0.8769 °C, respectively. Or, 28.8% and 36.0% below the single-electrode baseline (1.3695 °C). This declined with electrode count even though total active area was held constant. Also see Section 3.1 (Figure 4; Table 7). When the electrode count was fixed while varying the total active area (e.g., fixed-radius grid4 versus AM-Grid4; and fixed-radius grid9 versus AM-Grid9) a decline in stimulation-side ΔT was observed with increasing area. For example, the ΔT in grid4 decreased from 2.6913 °C to 0.9753 °C (63.8%), and in grid9 it decreased from 1.3400 °C to 0.8769 °C (34.6%). This two-point comparison is not designed to characterize the precise area-response relationship and is reported as a qualitative and exploratory finding (Section 4.3).

**TABLE 7:** Area-matched control series: total active area, stimulation-side maximum temperature rise, and ratio to the single-electrode reference under the representative stimulation condition ( I=50 mA, PW =700 μ s, F=10 Hz, t=300 s). Fixed-radius grid4 and grid9 are included for the complementary count-matched comparison (Section 3.2).

Configuration	Total active area $\left({\text{mm}}^{2}\right)$	Stim. max ΔT (°C)	Ratio to single
Single ( r=4 mm)	50.27	1.3695	1.000
AM-Grid4 ( N=4 , r=2 mm)	50.27	0.9753	0.712
AM-Grid9 ( N=9 , r=1.333 mm)	50.27	0.8769	0.640
Grid16 ( N=16 , r=1 mm)	50.27	0.7710	0.563
Grid4 (fixed radius, r=1 mm, for reference)	12.57	2.6913	1.965
Grid9 (fixed radius, r=1 mm, for reference)	28.27	1.3400	0.978

These results indicate that holding the total active electrode area constant does not eliminate the dependence of stimulation-side electrothermal load on electrode count.For example, both AM-Grid4 and AM-Grid9 continued to show substantial decline relative to the single-electrode reference. These results support contributions from both count-associated and area-associated geometric changes (Section 2.3.4) rather than active area governing the heating patterns reported in Sections 3.3 –Sections 3.5. The reported regime structure and $N^{*}$ crossover should be interpreted as reflecting the combined effect of electrode count and active area under the fixed 1 mm radius and 3 mm spacing geometry investigated here. We note that this analysis varied the total active area and electrode count while holding individual electrode shape fixed. Other parameters, such as the array footprint, inter-electrode spacing, perimeter, and active-region continuity were not varied against area and count.

### 3.3 Electrothermal regime characterization

Since individual electrode radius (1 mm) and inter-electrode spacing (3 mm) were held fixed by design while electrode count N was varied (Section 2.3.1), total active electrode area increases in direct proportion to N across the grid1-through-grid16 series $\left(N\times \pi r^{2}\right)$ , and array footprint increases correspondingly. The electrode count, total active area, and array footprint are therefore not independent variables within this primary series. This confound is directly addressed by the dedicated area-matched and count-matched control comparisons above (Section 3.2), which show that both count-associated and area-associated geometric changes contribute to the observed stimulation-side decline. The regime characterization presented below should accordingly be read as reflecting the combined action of both variables as they co-vary in this primary series, rather than electrode count in isolation.

**FIGURE 5:**
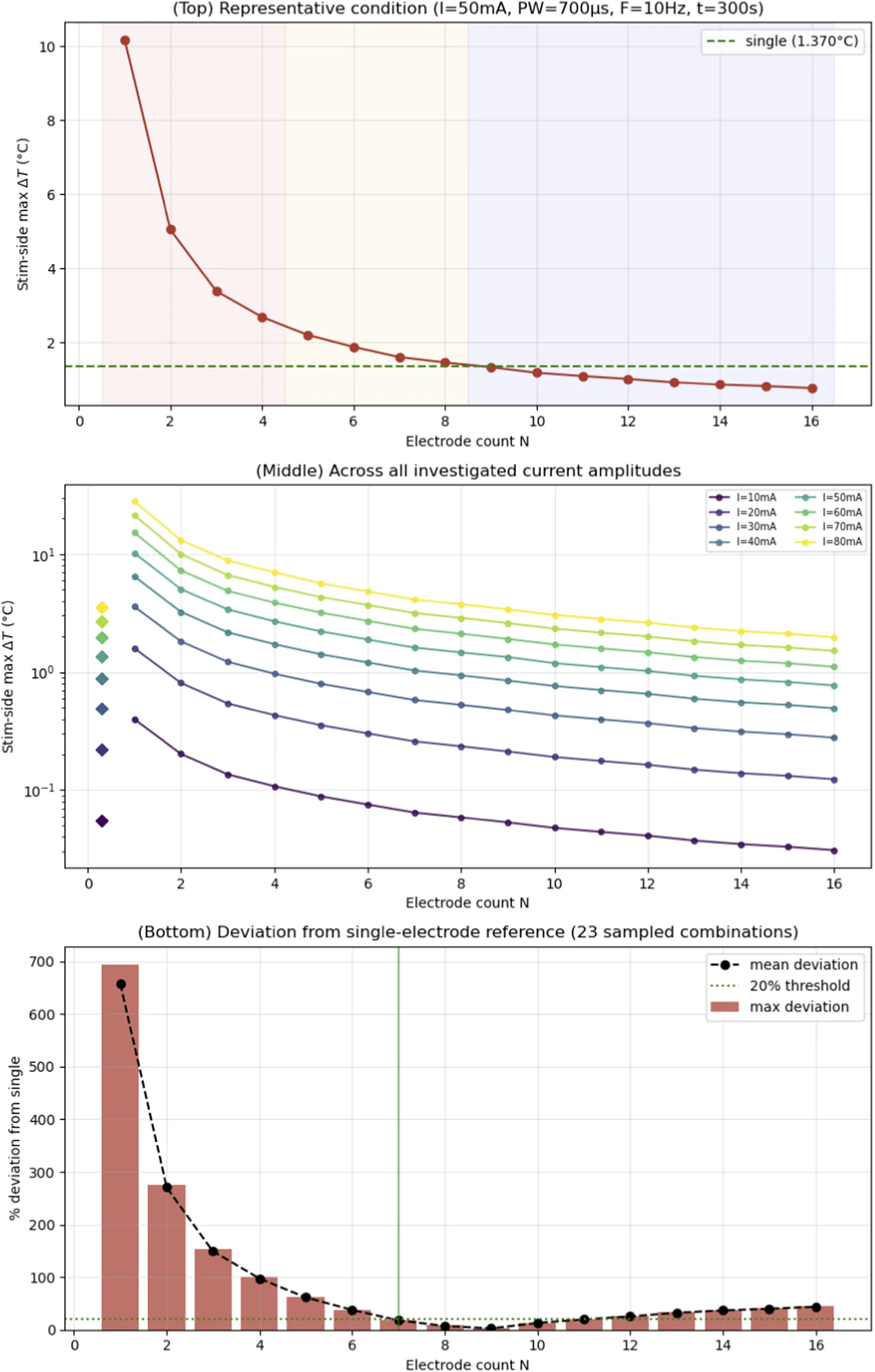
Electrothermal regime characterization (stimulation-side decomposition). (Top) Stimulation-side ΔT versus electrode count N at the representative condition ( I=50 mA, PW =700 μ s, F=10 Hz, t=300 s). Shaded regions indicate the three regimes described in the text; red dashed line: single-electrode reference ( ΔT=1.3695 °C). (Middle) Stimulation-side ΔT versus N across all investigated current amplitudes (PW =700 μ s, F=10 Hz, t=300 s); diamonds indicate the single-electrode reference at each amplitude. (Bottom) Deviation of stimulation-side ΔT from the single-electrode reference versus N , across 23 sampled ( I , PW, F ) combinations at t=300 s (Table 8). Bars: maximum deviation; dashed line: mean deviation. Green line: threshold $N_{20\%}=7$ (deviation first falls below 20%, not a convergence point; see text).

To characterize the relationship between electrode count and electrothermal behavior across the full subdivision range, peak current density and maximum temperature rise were evaluated as a function of N for grid1 through grid16 at the representative stimulation condition ( I=50 mA, PW =700 μ s, F=10 Hz, t=300 s). Results are shown in Figure 5.

Stimulation-side maximum temperature rise in the effective superficial skin layer declined continuously from grid1 ( ΔT=10.1807 °C) through grid16 ( ΔT=0.7710 °C), following an approximate power-law relationship with electrode count ( $\Delta T\approx 9.69\times N^{-0.91}$ , $R^{2}=0.999$ ; Section 3.1) rather than approaching and stabilizing at a plateau near the single-electrode baseline. Three regimes are nonetheless identified below for descriptive purposes, based on behavior relative to the single-electrode reference and the fixed return-electrode background contribution (Section 3.1).

- Extreme concentration regime ( N=1 –4): ΔT exceeded the single-electrode baseline substantially. Grid1 produced ΔT=10.1807 °C (7.43 × single); grid2 produced 5.0536 °C (3.69 × ); grid4 produced 2.6913 °C (1.97 × ).
- Transition regime ( N=5 –8): ΔT continued to decline, from 2.2100 °C (grid5, 1.61 × single) to 1.4663 °C (grid8, 1.07 × single), approaching but not crossing below the single-electrode reference.
- Background-dominated regime (N≥9) : Grid9 (1.3400 °C, 0.98 × single) marks the point at which stimulation-side ΔT first falls below the single-electrode reference. Rather than stabilizing near this baseline, ΔT continues declining through grid16 (0.7710 °C, 0.56 × single), falling further below both the single-electrode reference and the fixed return-electrode background contribution ( ≈ 1.38 °C; Section 3.1) as N increases further. The global metric’s apparent stabilization near the single-electrode baseline in this regime (Section 3.1) reflects the increasing dominance of this fixed background contribution rather than a genuine plateau in the stimulation array’s own behavior.

The point at which the stimulation-side deviation from the single-electrode reference first falls below 20% was re-examined using the stimulation-side decomposition. It evaluates the maximum percentage deviation across 23 sampled ( I , PW, F ) combinations at t=300 s for which stimulation-side data were extracted (Section 2.7). This point, denoted $N_{20\%}$ was used to distinguish it from the separate return-electrode background crossover $N^{*}$ discussed below, and occurred at $N_{20\%}=7$ . The maximum deviation was 18.99%, or just below the 20% criterion, while grid6 exceeded this criterion at 37.75%. We note that $N_{20\%}=7$ does not shrink monotonically beyond this point. The maximum deviation reaches a minimum at grid9 (3.23%) and a maximum at grid16 (44.28%) (Table 8). The deviation curve is better described as reaching a minimum near N=9 than as a plateau or convergence point beyond $N_{20\%}=7$ .

**TABLE 8:** Maximum and mean percentage deviation of stimulation-side ΔT from the single-electrode reference, evaluated across 23 sampled ( I , PW, F ) combinations at t=300 s.

Configuration	Max deviation (%)	Mean deviation (%)
Grid1	693.95	657.98
Grid2	274.80	270.71
Grid3	153.29	148.94
Grid4	100.21	97.23
Grid5	62.43	61.37
Grid6	37.75	37.62
Grid7	18.99	17.88
Grid8	8.21	7.17
Grid9	3.23	2.53
Grid10	13.11	13.09
Grid11	19.64	19.62
Grid12	25.68	25.35
Grid13	33.32	32.17
Grid14	37.32	36.70
Grid15	40.44	39.85
Grid16	44.28	43.73

### 3.4 Temperature evolution under pulsed stimulation

Transient temperature and thermal accumulation showed differences based on the active number of electrodes. In all electrode configurations, temperature increased rapidly during the initial stimulation phase and then rose more gradually thereafter. However, the shape of this transient differed qualitatively between grid1 and all other configurations. In the single, grid4, grid9, and grid16 configurations, the heating rate decreased monotonically and continuously throughout the full 300 s duration (e.g., single: from 0.0150 °C/s at t=0 –10 s to 0.00199 °C/s at t=290 –300 s), consistent with a standard, single-time-constant approach toward a quasi-steady thermal state. Grid1, in contrast, exhibited a non-monotonic transient even at 10 s temporal resolution (i.e., this is not an artifact of coarser sampling). The temperature rose to a local maximum of 9.616 °C at t=120 s, declined to a local minimum of 9.225 °C at t=210 s, and then rose again to the reported ΔT=10.1807 °C at t=300 s. The heating rate over this final segment increased rather than decreased (from 0.0054 °C/s at t=210 –220 s to 0.0152 °C/s at t=290 –300 s). Since this domain-wide maximum is evaluated as the maximum temperature anywhere in the model volume at each time step (Section 2.8), rather than at a single fixed material point, a decline followed by an accelerating rise in this metric is consistent with the maximum-temperature location physically migrating between two spatially distinct thermal regimes. In other words, an initial low-thermal-mass region immediately beneath the 1 mm electrode that heats and locally overshoots quickly before being limited by strong lateral heat loss into the surrounding tissue, and a second longer-time-constant region that continues to accumulate heat and eventually exceeds the first location’s declining temperature, becoming the new domain maximum. This interpretation is developed further in Section 4.1. Grid1 accordingly does not approach a quasi-steady thermal state within the simulated 300 s duration, unlike the other four configurations, and the reported grid1 ΔT should be interpreted as a t=300 s snapshot value rather than an asymptotic steady-state value.

**FIGURE 6:**
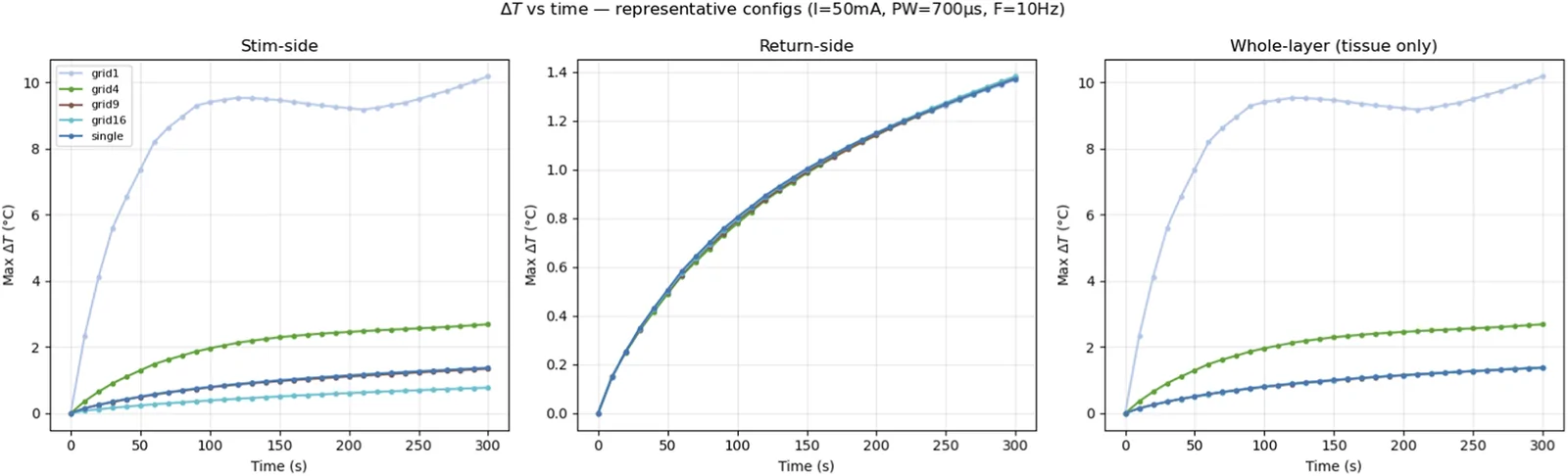
Temperature rise versus time for representative electrode configurations under representative stimulation conditions ( I=50 mA, PW =700 μ s, F=10 Hz). (Left) Stimulation-side maximum ΔT . (Middle) Return-side maximum ΔT . (Right) Whole-layer (tissue-only) maximum ΔT , effective superficial skin layer. The non-monotonic grid1 transient (Section 3.4) is visible in the stimulation-side and whole-layer panels but not in the smooth, monotonic return-side panel, consistent with this transient feature originating within the stimulation-side region rather than from migration toward the return electrode (Section 4.1).

Images in Figure 6 are shown for the configuration using I=50 mA, PW =700 μ s, and F=10 Hz. Here, grid1 produced the highest temperature rise (10.1807 °C) in both the effective superficial skin layer and the dermis throughout the stimulation duration. This is consistent with the stronger superficial current crowding identified in Section 3.1. In contrast, the single (1.3695 °C), grid9 (1.3400 °C), and grid16 (0.7710 °C) configurations show progressively lower stimulation-side heating rather than similar heating; the close numerical agreement among these three configurations under the global evaluation domain (Section 3.1) reflects the fixed return-electrode background contribution rather than genuinely similar stimulation-side heating, as discussed further in Section 4.3.

### 3.5 Parameter-dependent thermal characteristics

**FIGURE 7:**
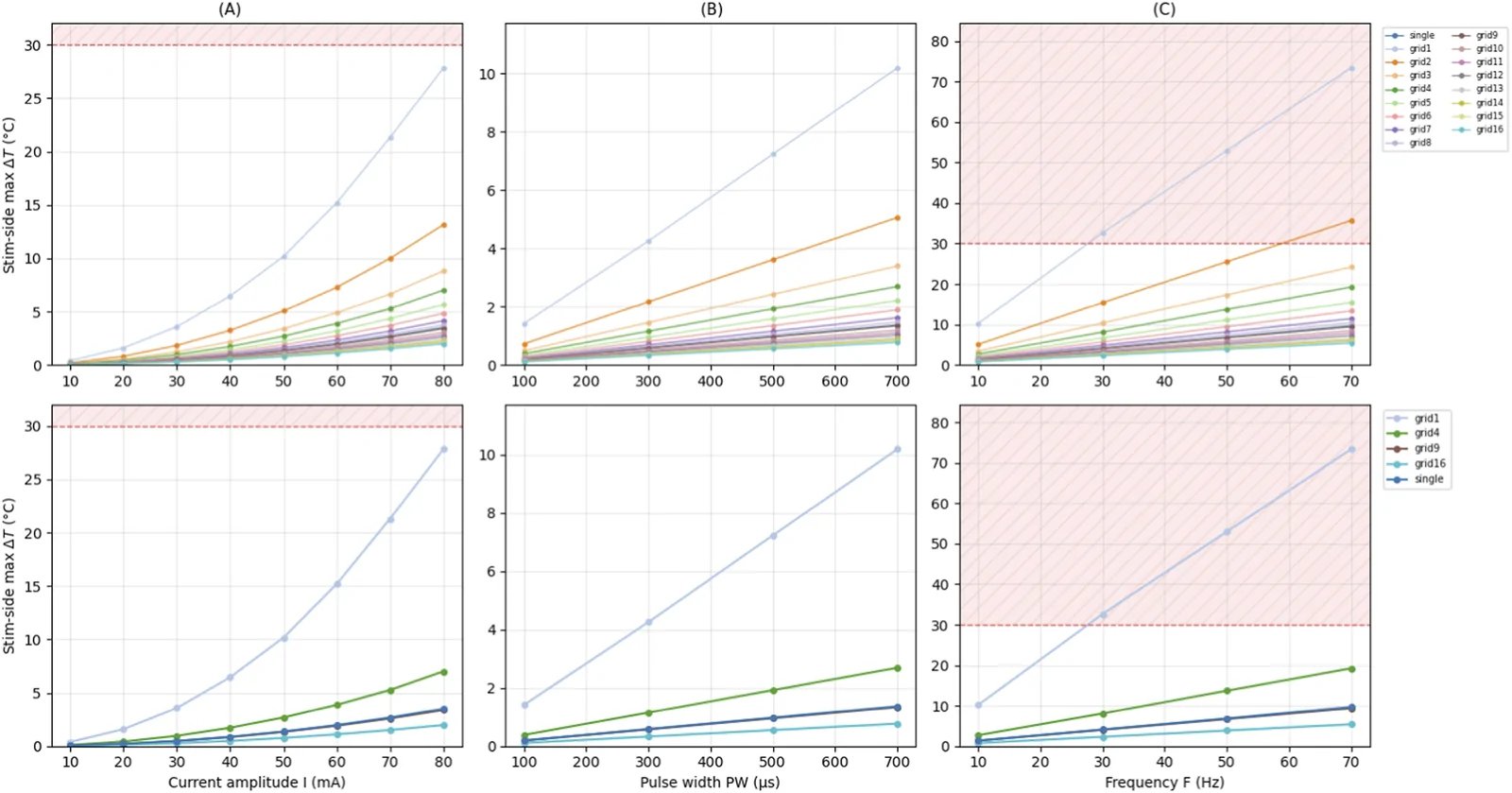
Stimulation-side maximum ΔT across the three investigated stimulation parameters, t=300 s. **(A)** Current amplitude (PW =700 μ s, F=10 Hz). **(B)** Pulse width ( I=50 mA, F=10 Hz). **(C)** Stimulation frequency ( I=50 mA, PW =700 μ s). (Top row) All electrode configurations (grid1–grid16 and single). (Bottom row) Representative configurations (grid1, grid4, grid9, grid16, single). In all panels, the horizontal dashed red line at ΔT=30 °C indicates the model-invalid regime in which temperature-independent material properties and the perfusion-free assumption are no longer physically defensible (Section 4.3); data above this line are shown for parameter-space completeness but should not be interpreted as physically accurate absolute temperatures.

The effects of stimulation current amplitude (Figure 7), pulse width, and stimulation frequency on maximum temperature rise were evaluated across all electrode configurations. The relative ordering among configurations remained consistent with the observations reported in Sections 3.1 and 3.3. That is, configurations in the extreme concentration regime (N≤4) consistently produced the highest temperature rises, while background-dominated regime configurations (N≥9) declined in stimulation-side ΔT with increasing N . Because several stress-test parameter combinations in this sweep produce temperature rises well beyond the range over which the present temperature-independent, perfusion-free model remains physically defensible—approximately ΔT>30 °C, above which real tissue electrical/thermal conductivity, perfusion, and protein structure are known to change substantially with temperature (Section 4.3)—this model-invalid region is shaded in Figure 7 below to visually distinguish it from the physically interpretable regime; data points within the shaded region are retained (rather than truncated) to preserve the full comparative parameter-space picture, but should not be read as physically accurate absolute temperature predictions.

#### 3.5.1 Influence of current amplitude

Temperature rise increased with stimulation current amplitude across all electrode configurations (Figure 7A). At I=10 mA (PW =700 μ s, F=10 Hz), stimulation-side ΔT was 0.3973 °C for grid1 versus 0.0548 °C for single (7.25 × ), while grid9 (0.0530 °C, 0.97 × single) and grid16 (0.0308 °C, 0.56 × single) had already separated below the single-electrode reference. At I=80 mA, grid1 produced ΔT=27.8298 °C (absolute maximum temperature T=64.83 °C, given the $T_{0}=37$ °C initial condition defined in Section 2.8) compared with 3.5061 °C for single (7.94 × ); 7.0144 °C for grid4 (2.00), 3.3930 °C for grid9 (0.97 × =) and 1.9737 °C for grid16 (0.56 × ). This shows a ratio structure that is invariant to current amplitude across the 8-fold investigated range. The grid9/single and grid16/single ratios varied by less than 1% between I=10 and I=80 mA. This is consistent with the near-linear ΔT scaling established in Section 3.5.4.

#### 3.5.2 Influence of pulse width

Increasing pulse width produced a consistent increase in stimulation-side ΔT across all electrode configurations (Figure 7B), reflecting the proportionally greater energy delivered per unit time as pulse duration increased. At PW =100 μ s ( I=50 mA, F=10 Hz), grid1 produced ΔT=1.4187 °C versus 0.1956 °C for single (7.25 × ), while grid9 (0.1914 °C, 0.98 × single) and grid16 (0.1101 °C, 0.56 × single) were already separated from the single-electrode reference and from each other. This ratio structure was unchanged across the full PW =100 –700 μ s range investigated. Grid1 maintained the highest temperature rise throughout the investigated pulse-width range, while grid9 through grid16 remained well separated from the single-electrode baseline and from each other, rather than converging toward it. As with the current-amplitude sweep (Section 3.5), several of the higher-frequency, longer-pulse-width combinations investigated here drive grid1 (and, to a lesser extent, grid2–grid4) into the model-invalid region ( ΔT>30 °C, shaded in Figure 7B). These upper-range combinations should accordingly be read as stress-test conditions (Section 2.7) rather than physically accurate absolute temperature predictions.

#### 3.5.3 Influence of stimulation frequency

Temperature rise increased with stimulation frequency across all electrode configurations (Figure 7C), reflecting enhanced cumulative energy deposition and reduced inter-pulse cooling intervals during prolonged stimulation. At F=70 Hz ( I=50 mA, PW =700 μ s), grid1 produced ΔT=73.3555 °C versus 9.6865 °C for single (7.57 × ), while grid9 (9.3832 °C, 0.97 × single) and grid16 (5.3968 °C, 0.56 × single) remained clearly separated from the single-electrode baseline and from each other across the investigated frequency range, rather than converging toward it or toward one another. As in the current-amplitude and pulse-width sweeps above, the highest-frequency, longest-pulse-width combinations drive grid1 well into the model-invalid region ( ΔT>30 °C, shaded in Figure 7C); these combinations constitute stress tests of the parameter space (Section 2.7) rather than physiologically representative operating conditions, and the corresponding ΔT values should be interpreted accordingly (Section 4.3).

#### 3.5.4 Energy-proxy scaling analysis

Since the model uses temperature-independent material properties and Joule heating scales as $\sigma |E{|}^{2}\propto I^{2}$ , the stimulation-side maximum temperature rise was analyzed against an energy-proxy scaling variable defined as $I^{2}\times PW\times F$ , rather than considering current amplitude, pulse width, and frequency as independent effects only. This proxy is used here as a convenient, monotonic comparative scaling variable for the regression rather than as a literal reproduction of the duty cycle applied in the simulation itself (which additionally includes a biphasic factor of 2, 2×PW×F ; Section 2.5.2); because this is a constant multiplicative factor common to all configurations and stimulation conditions, it does not affect the fitted exponents n or the between-configuration k -ratios reported below, only the absolute scale of k . For five representative electrode configurations (single, grid1, grid4, grid9, grid16), stimulation-side ΔT was regressed against this proxy using a power-law fit $\left(\Delta T=k\cdot {proxy}^{n}\right)$ across the 23 sampled ( I , PW, F ) combinations at t=300 s described in Section 3.3, a partial rather than the complete 128-combination sweep.

The fitted exponent n was close to 1 for single, grid4, grid9, and grid16 (range: 1.0000–1.0037, $R^{2}\geq 0.9998$ ), consistent with near-linear electrothermal coupling under the temperature-independent, perfusion-free model assumptions (Section 2.5). Grid1 showed a linear exponent ( n=1.0230 , $R^{2}=0.9987$ , the lowest fit quality among the five configurations), consistent with grid1’s previously established non-equilibrium transient behavior (Section 3.4). This suggests mild systematic deviation from a pure power-law fit at a fixed t=300 s snapshot. Additionally, the fitted proportionality constant k differed substantially across configurations. For example, it was 5.2 when comparing grid1 and the single-electrode reference, and then falling to 0.96 and 0.57 when compared to grid9 and grid16.This is consistent in direction and order of magnitude with the stimulation-side ΔT ratios reported at the representative condition (Table 9). However, the discrepancy for grid1 reflects the joint trade-off between k and n in the fit given grid1’s non-equilibrium behavior. These results indicate that no single configuration-independent scaling curve describes all seventeen configurations; rather, each configuration’s stimulation-side response follows its own approximately linear energy-proxy scaling. This suggests configuration-dependent proportionality constant k that reflects the electrode-count- and area-dependent decline established in Section 3.3 (Table 10).

**TABLE 9:** Quantitative comparison of stimulation-side maximum temperature rise, absolute maximum temperature ( $T=T_{0}+\Delta T$ , given the uniform $T_{0}=37$ °C initial condition; Section 2.8), and stimulation-side 99th-percentile current density ( $J_{\mathrm{p99}}$ , both nodal and area-weighted formulations) under representative stimulation conditions ( I=50 mA, PW =700 μ s, F=10 Hz), including absolute values and relative ratios normalized to the single-electrode baseline. Both ΔT and $J_{\mathrm{p99}}$ are evaluated within the stimulation-side region of interest defined in Section 2.8, with the corresponding return-side and global values reported in Table 6 and Section 3.1. The nodal formulation of $J_{\mathrm{p99}}$ is reported as the primary current-density metric throughout this study, for the reasons discussed in Sections 2.8 and 3.1; the area-weighted formulation is reported alongside it here to demonstrate that the comparative ranking and regime-relevant conclusions reported in this study do not depend on this formulation choice (Section 3.1). A direct $J_{\text{max}}$ versus $J_{\mathrm{p99}}$ comparison is provided in Figure 3.

Configuration	Max ΔT (°C)	Absolute max T (°C)	Ratio to single	Stim. $J_{\mathrm{p99}}$ , nodal (A/m^2^)	Ratio to single	Stim. $J_{\mathrm{p99}}$ , area-wt. (A/m^2^)	Ratio to single
Single	1.3695	38.37	1.000	442.4	1.000	340.4	1.000
Grid1	10.1807	47.18	7.434	859.5	1.943	668.7	1.964
Grid4	2.6913	39.69	1.965	640.9	1.449	481.1	1.413
Grid9	1.3400	38.34	0.978	419.4	0.948	322.1	0.946
Grid16	0.7710	37.77	0.563	299.7	0.677	267.7	0.786

**TABLE 10:** Power-law fits of stimulation-side maximum temperature rise against the energy proxy $I^{2}\times PW\times F$ , for five representative configurations, across the 23 sampled ( I , PW, F ) combinations at t=300 s described in Section 3.3 (a partial rather than the complete 128-combination sweep). $\Delta T=k\cdot {proxy}^{n}$ ; k ratios are broadly, though not exactly, consistent with the stimulation-side ΔT ratios reported in Table 9 at the single representative condition, with grid1’s larger deviation attributable to its non-equilibrium transient behavior (Section 3.4).

Configuration	k	n	$R^{2}$	k ratio to single
Single	$7.759\times 10^{-8}$	1.0005	0.999946	1.00
Grid1	$4.037\times 10^{-7}$	1.0230	0.998705	5.20
Grid4	$1.451\times 10^{-7}$	1.0037	0.999831	1.87
Grid9	$7.436\times 10^{-8}$	1.0016	0.999983	0.96
Grid16	$4.405\times 10^{-8}$	1.0000	1.000000	0.57

#### 3.5.5 Comparative summary

Across all three stimulation parameters, grid1 produced the highest maximum temperature rise at all investigated parameter levels. The complete regime ordering—extreme concentration (N≤4) , transition ( N=5 –8), and background-dominated (N≥9) —was preserved across all parameter combinations. Quantitative comparison under the representative stimulation condition is provided in Table 9.

### 3.6 Thermal transition boundary analysis

**FIGURE 8:**
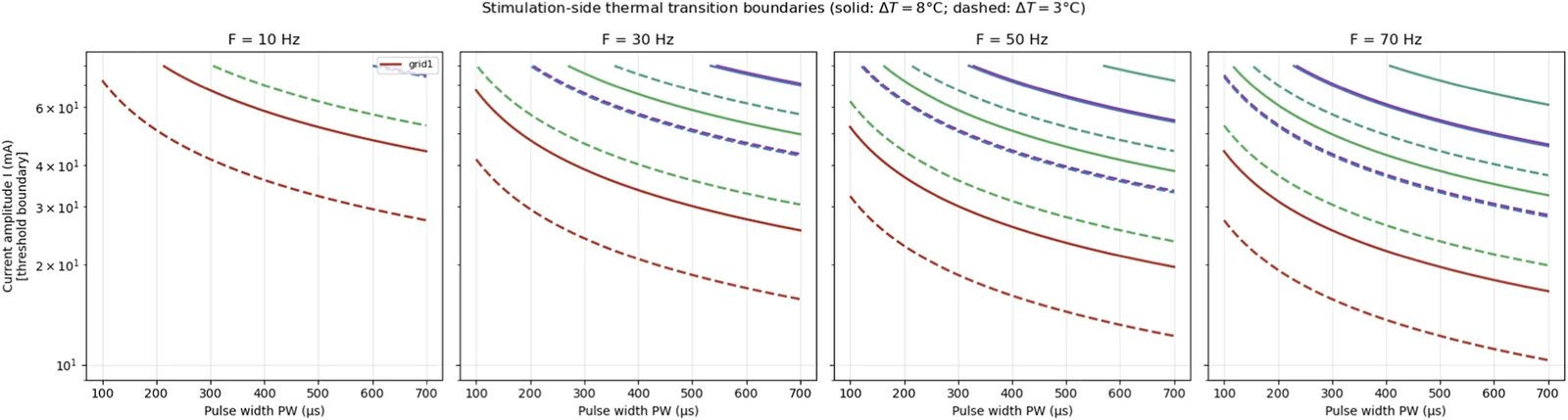
Stimulation-side thermal transition boundaries in the current-amplitude/pulse-width parameter space, shown separately for each of the four investigated stimulation frequencies. Solid lines indicate ΔT=8 °C boundaries; dashed lines indicate ΔT=3 °C boundaries, for grid1, grid4, single, grid9, and grid16. Boundaries were computed analytically from the stimulation-side energy-proxy power-law fits established for these five representative configurations (Table 10; Section 3.5.4) rather than from a full re-sweep of the stimulation-side decomposition across the complete parameter grid. Curve segments falling outside the investigated current-amplitude range (10–80 mA) are not shown.

Thermal transition boundaries (Figure 8) were evaluated across the stimulation-parameter space defined by current amplitude, pulse width, and stimulation frequency, using thresholds of ΔT=3 °C and ΔT=8 °C. Using the stimulation-side energy-proxy fits established for the five representative configurations (Table 10), the current amplitude required to reach the ΔT=8 °C threshold at fixed PW =700 μ s, F=10 Hz was estimated for each configuration. Specifically, grid1 reached this threshold at the lowest current amplitude ( I≈44 mA), followed by grid4 ( I≈86 mA), single and grid9 were at closely similar amplitudes ( I≈121 and 123 mA, respectively, consistent with grid9’s close correspondence to the single-electrode reference established in Section 3.3), while grid16 was at a higher amplitude ( I≈161 mA) than either single or grid9. This ordering suggests that grid16 requires significantly more current than single or grid9 to reach the same threshold. This observation is also consistent with the stimulation-side decline established across the grid9-through-grid16 range (Section 3.3).

Parameter-space coverage was quantified as a discretized count of the number of sampled ( I , PW, F ) combinations satisfying each temperature-rise criterion across the 128 investigated combinations (e.g., 8 current levels × 4 pulse-width levels × 4 frequency levels per Section 2.7). Defining three regions: ΔT<3 °C, 3≤ΔT<8 °C, and ΔT≥8 °C (Section 3.1). Since no interpolation between sampled points was performed, the reported counts and ratios (e.g., the 4.50 × value for grid1 below) reflect this specific sampling resolution rather than a continuous volume. The sensitivity of this count-based ratio to the density and range of the sampled ( I , PW, F ) grid was not directly tested in the present study (e.g., by repeating the analysis on a finer or coarser sampling grid). It is expected that the ratio is sensitive to this change but is left for future work.

The parameter-space analysis evaluated using the stimulation-side decomposition across the 128-combination sweep for all seventeen configurations revealed a declining relationship between electrode count and the ΔT≥8 °C region. Grid1 occupied 72 of 128 combinations in the high-temperature region (4.50 × single), grid2 occupied 50 combinations (3.13 × single), grid4 occupied 34 combinations (2.13 × single), and grid8 occupied 18 combinations (1.13 × single). This is consistent with the global-metric-based counts at sparse configurations, where the stimulation side dominates the signal (Section 3.1). From grid9 onward, however, the stimulation-side count continues to decline rather than remaining fixed at a common value. For example, grid9 and grid10 each occupy 14 combinations (0.875 × single), and fall to 12 (grid11), 8 (grid12–13), and finally 4 combinations for grid16 (0.25 × single). This corresponds to a fourfold reduction beyond grid9. This confirms the same power-law-like decline in stimulation-side electrothermal load established for ΔT and $J_{\mathrm{p99}}$ in Sections 3.3 and 3.1, and provides further evidence that global-metric plateauing reflects the fixed background contribution rather than genuine saturation. The discretized parameter-space count summary is provided in Table 11.

**TABLE 11:** Summary of stimulation-side discretized parameter-space counts (out of 128 sampled combinations; no interpolation) for the three temperature-rise regions across all electrode configurations. See Section 2.7 for the discretization noted above.

Config.	ΔT<3 °C	3≤ΔT<8 °C	ΔT≥8 °C	Total	Ratio ( ≥ 8 °C)
Single	89	23	16	128	1.000
Grid1	35	21	72	128	4.500
Grid2	48	30	50	128	3.125
Grid3	61	29	38	128	2.375
Grid4	69	25	34	128	2.125
Grid5	72	30	26	128	1.625
Grid6	78	26	24	128	1.500
Grid7	83	24	21	128	1.312
Grid8	87	23	18	128	1.125
Grid9	89	25	14	128	0.875
Grid10	92	22	14	128	0.875
Grid11	94	22	12	128	0.750
Grid12	94	26	8	128	0.500
Grid13	100	20	8	128	0.500
Grid14	101	20	7	128	0.438
Grid15	102	19	7	128	0.438
Grid16	102	22	4	128	0.250

### 3.7 Numerical robustness evaluation

The numerical robustness of the coupled electrothermal simulation framework was assessed through mesh-convergence analysis, domain-size verification, and physical consistency checks, as described in Section 2.9. The results of these evaluations are summarized here to support the reliability of the comparative findings reported in Sections 3.1 through 3.6.

**FIGURE 9:**
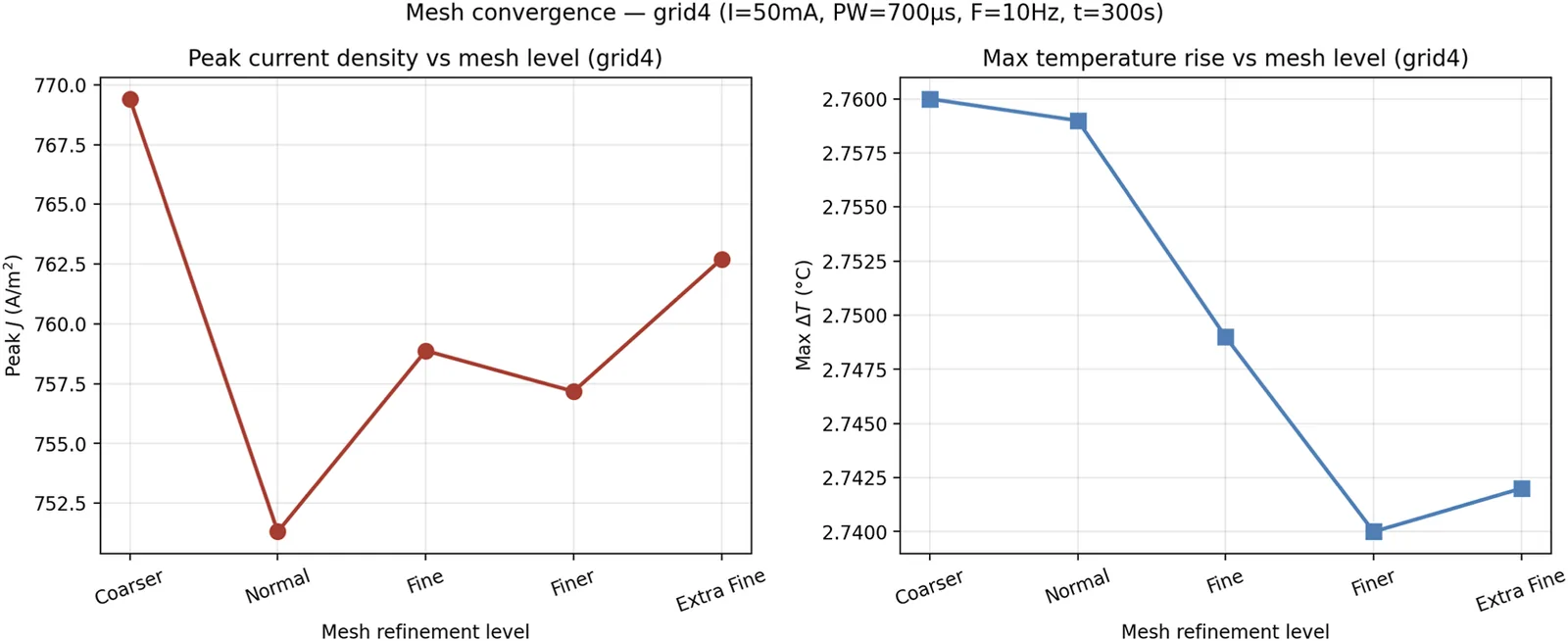
Mesh-convergence analysis showing peak current density and maximum temperature rise as a function of mesh refinement level for the grid4 configuration under representative stimulation conditions ( I=50 mA, PW =700 μ s, F=10 Hz, t=300 s).

Mesh-convergence analysis demonstrated stable electrothermal simulation behavior across progressively refined mesh conditions (Figure 9). Variations in maximum temperature rise near the electrode-tissue interface remained below the 2% convergence criterion defined in Section 2.9 at every refinement step. The raw peak current density likewise varied by 2.4% across all five mesh levels. and changed non-monotonically rather than approaching a single limit from one side. This reflects the sampling sensitivity of the electrode-edge singularity rather than an unconverged mesh, as discussed in detail in Section 2.9. The three-regime ordering of electrothermal behavior remained unchanged across all tested mesh refinement levels. This was true across all three regimes–i.e., N≤4 , N=5 –8, N≥9 .

Domain-size verification demonstrated negligible changes in current-density distribution and maximum temperature rise near the stimulation region after enlargement of the computational tissue domain from 150 × 150 ${\text{mm}}^{2}$ to 250 × 250 ${\text{mm}}^{2}$ . The observed differences in both metrics were less than 1%, supporting the use of the 150 × 150 ${\text{mm}}^{2}$ domain size for the comparative analysis.

Collectively, the mesh-convergence, domain-size, and physical consistency results support the numerical stability and physical validity of the comparative electrothermal modeling framework.

## 4 Discussion

The findings reported below should be interpreted as qualitative, geometry-specific comparative modeling trends obtained under the idealized assumptions described in Section 2, rather than as validated thermal safety predictions. This framing applies throughout the following interpretation and design-implication discussion and is revisited explicitly in Section 4.3.

### 4.1 Interpretation of electrothermal regime behavior and underlying mechanisms

This study demonstrated that electrothermal behavior did not increase monotonically with active electrode number under the fixed electrode geometry conditions. Instead, stimulation-side ΔT declined with electrode count following an approximate power-law relationship (Section 3.1), without converging toward or stabilizing at the conventional single-electrode configuration’s behavior. Conversely, the grid1 configuration produced the highest superficial current density and temperature rise among the investigated configurations.

The elevated heating observed in grid1 reflects an expected consequence of localized current crowding due to a smaller active electrode region. This is consistent with well-established small-electrode current-concentration effects reported in prior electrode-tissue interface literature (RaviChandran et al. 2020; Datta et al., 2008; Khadka and Bikson, 2020), rather than a novel finding of the present study. Under the idealized current-controlled conditions of the present model, the sparse distribution of active electrodes concentrated current flow near individual electrode sites, producing stronger superficial Joule heating (Figure 6) and earlier transition into high-temperature-rise.

This concentrated current pathway also offers a plausible explanation for the qualitatively distinct transient signature reported for grid1 in Section 3.4. Specifically, the local temperature maximum near t=120 s, the subsequent decline to t≈210 s, and the accelerating late-phase rise through t=300 s. Because grid1 combines the smallest active electrode area with the highest current concentration among the investigated configurations, it is expected to have the largest disparity between thermal surface responses directly beneath the electrode. Specifically, it demonstrated a dip and then a sharp rise in temperature. This was tracked in spatial coordinates across simulated intervals (e.g., at t=100 , 150, 210, and 300 s) to verify that the reported maximum is attributable to two distinct spatial locations rather than a single point. A stimulation-side/return-side/global decomposition of the transient temperature field (Section 3.1) confirmed that this non-monotonic dip-then-rise occurred within the stimulation-side region of interest. Thus, it was not attributable to the domain-wide maximum transferring between the stimulation and return electrodes. It was also found that the return-side transient over the same interval was smooth and monotonic. However, some caution should still remain when observing the region immediately beneath the electrode and the surrounding tissue volume. None of the other sixteen investigated configurations exhibited this non-monotonic transient signature, suggesting distributed heat profiles.

A central finding of this study is that the fixed-radius grid1-through-grid16 progression does not approach or remain at a stable plateau as electrode count increases. Although grid9 and grid16 contained more electrode elements and a greater total active area, their stimulation-side electrothermal behavior was lower than that of the single-electrode reference (Section 3.3). The close numerical agreement between single, grid9, and grid16 reflects an increased influence of the fixed return-electrode (Section 3.1), rather than a plateau in the stimulation array’s own electrothermal behavior.

In the case of the stimulation-side, $J_{\mathrm{p99}}$ saw lower current density from grid9 (419.4 A/m^2^) to grid16 (299.7 A/m^2^). This is a decline of approximately 29% and is consistent with the stimulation-side power decline established across the full grid1–grid16 range (Section 3.1). Similarly, using a fixed electrode radius and total current, the nominal current density per-contact at the electrode terminal, $I_{\text{total}}/\left(N\pi r^{2}\right)$ , scaled with a ratio of 1/N . The data in Table 2 demonstrate this, where the ratio of nominal current density between grid16 and grid9 is 994.7/1768.4=0.5625 . This matches a ratio of 9/16 to four significant figures. On the observed stimulation-side, the $J_{\mathrm{p99}}$ declined by 29% reflecting an attenuation at a rate of 1/N due to current spreading in the hydrogel and superficial tissue layers. The stimulation-side decline is also reflected in the corresponding ΔT comparison. Here, grid9 declines from 1.3400 °C to 0.7710 °C for grid16 (42.5%) per Table 9. This is consistent in direction and magnitude with the $J_{\mathrm{p99}}$ decline, indicating an apparent convergence toward the single-electrode baseline (Section 3.1).

This interpretation is nuanced by the layer-dependent current-density behavior described in Section 3.1. Generally, the results appear to be governed by edge-singularity sampling (Section 2.9) rather than from topology-driven effects. However, at deeper-layers the features may change. For example, the dermis-layer grid10 has a higher $J_{\mathrm{p99}}$ metric on the stimulation-side $J_{\mathrm{p99}}$ and does not appear to be related to raw-maximum sampling noise. This suggests a layer- and footprint-specific effect that is distinct from the trend established for the superficial-layer or ΔT -based changes Section 3.3.

This also highlights the distinction between the stimulation interface dimensions and effective electrothermal stimulation area. Although the configurations shared identical return-electrode geometry and stimulation conditions, their active electrode regions were spatially different. This resulted in varying electrothermal responses. In the grid1 configuration, the active region was smaller, so the effective electrothermal loading was higher at the superficial layer. In contrast, the grid9 and grid16 configurations produced more spatially distributed configurations, causing the electrothermal stimulation area to also increase.

Collectively, this indicates that stimulation-side electrothermal load is collectively shaped by electrode count and total active area (Section 3.2) rather than by either variable individually. Scaling the number of electrodes, the total active area, or both can therefore be used to modulate the current-density and heating properties of the interface. This also indicates that sparse electrode configurations amplify localized superficial heating substantially, whereas increased electrode count and/or active area together drives a continued, non-plateauing decline in stimulation-side load.

This co-dependence is directly demonstrated by the dedicated area- and count-matched control comparisons (Section 3.2). When electrode radius was increased to hold total active area constant, both the N=4 and N=9 area-matched configurations showed stimulation-side declines relative to the single-electrode reference (28.8% and 36.0% below baseline, respectively). Thus, the total active area was not sufficient to explain the fixed-radius series’ behavior. Conversely, when using a fixed electrode count but varied areas, it demonstrated a decline with increasing area. Together, these results indicate that both count-associated and area-associated geometric changes contribute to the electrothermal regimes reported.

### 4.2 Implications for HD-Inspired neurostimulation design

The present study provides electrothermal modeling insight directly relevant to HD-inspired surface neurostimulation systems. Previous distributed-electrode studies have focused primarily on electric-field focality, current steering, and stimulation selectivity (Datta et al., 2008; Villamar et al., 2013; Datta et al., 2009; Guler et al., 2016; Jain et al., 2023; Mancabelli et al., 2026). However, comparatively few studies have examined how array electrodes affect electrothermal behavior. The results presented herein support HD-style electrode modeling work by evaluating spatial dependencies on the electrothermal effects.

Rather than converging toward a continuous-electrode-like plateau, stimulation-side electrothermal load continued to decline while electrode count (and active area) increased beyond grid9 (Section 3.3). In particular, dense HD-inspired arrays can reduce localized superficial current density and heating beyond that of conventional single electrode with matching areas. This trend was shown in both the current-density (Section 3.1) and ΔT -based (Section 3.3) stimulation-side comparisons. It is relevant for HD-inspired wearable neurostimulation interfaces with large numbers of electrodes and/or programmable active areas to influence current controllability and spatial targeting. It also indicates that the electrothermal benefit of electrode subdivision does not saturate at a single value but can be scaled.

Notably, sparse electrode subdivisions within a compact stimulation interface may unintentionally elevate localized electrothermal loading. As such, a key variable in the design of these HD stimulation arrays is the optimization of electrode density within the grid, along with the designed active area. For example, grid4 showed elevated-temperature-rise characteristics at substantially lower stimulation settings, suggesting that sparse subdivision levels such as those represented by grid4 may constitute a less favorable design regime under the present idealized assumptions. Specifically, the electrode count was insufficient to substantially dilute per-electrode current density, concentrating current into localized hotspots instead. In the present study, stimulation-side deviation from the single-electrode reference first fell below a 20% threshold at $N_{20\%}=7$ and reached a minimum near N=9 (Section 3.3). However, this minimum does not represent a sustained plateau. Instead, the stimulation-side load continued to decline, and deviation from the single-electrode reference increased through grid16. These thresholds are specific to the 1 mm electrode radius, 3 mm spacing, hydrogel geometry, and reference-electrode configuration investigated here, and should be interpreted as geometry-specific modeling observations rather than general design rules for HD electrode arrays.

Future HD-inspired stimulation designs should jointly consider electrode count, total active area, and array footprint, rather than treating any single variable in isolation as the primary design lever. The present comparative electrothermal framework may serve as a useful reference for evaluating the electrothermal consequences of electrode subdivision strategies in compact and wearable HD-inspired surface neurostimulation systems.

### 4.3 Limitations

The present model provides qualitative, geometry-specific comparative electrothermal trends under a set of idealized modeling assumptions. It has not been experimentally validated against phantom, *ex vivo*, or *in vivo* thermal measurements. Accordingly, the reported regimes, thresholds, and magnitudes should not be interpreted as validated thermal safety predictions, and several specific idealizations further bound the conditions under which the present comparative trends apply.

There are several limitations in this study that should be acknowledged. First, the computational model employed a simplified multilayer planar tissue geometry with homogeneous and isotropic material properties. Although this approach enabled controlled comparative analysis across electrode configurations, real biological tissues exhibit anatomical curvature, structural heterogeneity, vascular variability, and subject-dependent physiological differences that may alter local current-density distribution and electrothermal behavior. The isotropic muscle assumption warrants particular caution, as skeletal muscle conductivity is direction-dependent and fiber orientation relative to the electrode pair could influence current penetration depth and deeper current-distribution patterns. This assumption was not tested via sensitivity analysis in the present study. Relatedly, the domain-size verification described in Section 2.9 tested only the lateral extent of the tissue domain (150 × 150 ${\text{mm}}^{2}$ versus 250 × 250 ${\text{mm}}^{2}$ ). The total tissue depth, the 10 mm muscle-layer thickness, the electrically insulating bottom boundary, and the fixed 37 °C thermal bottom boundary (Section 2.5.3) were held fixed throughout and were not varied as part of this verification. Since deeper current penetration could in principle be sensitive to muscle-layer depth and to these bottom boundary conditions, this represents an additional untested assumption alongside the muscle anisotropy limitation discussed above. The sensitivity analysis of tissue depth and bottom electrical/thermal boundary conditions is identified as a direction for future work. The present results should therefore be interpreted as comparative computational trends rather than subject-specific predictions.

Second, a further simplification concerns the electrode-tissue interface. The present model assumes ideal electrical continuity at all electrode-electrolyte and electrolyte-tissue boundaries (Section 2.4.2), with no explicit representation of contact impedance, electrode polarization, capacitive double-layer effects, skin hydration variability, pressure-dependent contact quality, sweat accumulation, hydrogel degradation, or spatially local impedance heterogeneity. These interfacial factors are known to substantially influence current crowding and superficial heating in real transcutaneous stimulation scenarios. Thus, their omission represents a central limitation of the present idealized framework rather than a minor simplification.

This simplification directly affects the interpretation of the main results in two ways. First, the absolute magnitude of the reported current densities and temperature rises should be understood as governed entirely by tissue-layer conduction under perfect interfacial contact. The real-world contact non-idealities are likely to alter these magnitudes, and may do so disproportionately for the sparse, small-area grid1 configuration, where local pressure and hydration variability could amplify the already-concentrated current pathways to an extent not captured here. Second, because interfacial idealization was applied uniformly across all seventeen configurations, the relative comparative trends reported here—including the three-regime structure and the $N_{20\%}=7$ threshold—are expected to be more robust to this simplification than the absolute ΔT and peak- J values. Nonetheless, quantitative real-world electrode-skin safety predictions cannot be directly derived from the present idealized-interface model, and sensitivity-analysis-based or experimental characterization of contact-impedance effects (e.g., via a range of hydrogel/skin contact impedance values) represents an important direction for future work.

**FIGURE 10:**
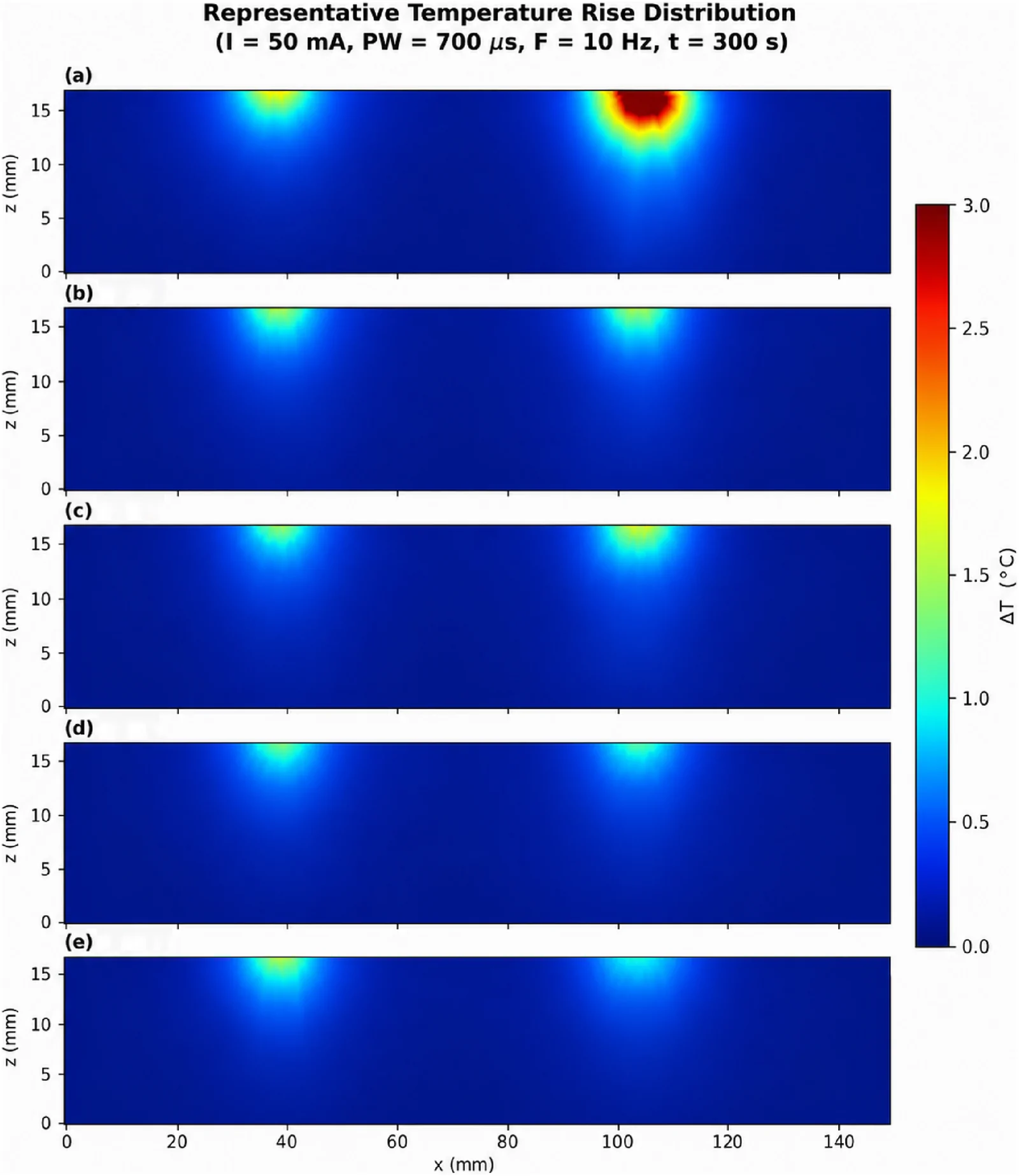
Representative temperature-distribution maps for all electrode configurations at end of stimulation ( t=300 s; I=50 mA, PW =700 μ s, F=10 Hz). Cross-sectional view along the central longitudinal axis of the stimulation electrode array, restricted to the tissue domain (excluding the electrode and hydrogel/electrolyte layers above the tissue surface). **(a)** Grid1, **(b)** Single, **(c)** Grid4, **(d)** Grid9, **(e)** Grid16. All panels share a common manual color-table range (0 °C–3.0 °C) so that grid1 (stimulation-side ΔT=10.18 °C) can be included on the same shared colorbar as the other configurations; grid1’s hotspot saturates at the top of the color scale, directly illustrating its substantially larger and more spatially concentrated temperature rise relative to the other configurations shown here.

Third, the model assumes temperature-independent electrical and thermal material properties throughout the tissue domain (Section 2.6), where tissue perfusion was not included in the bioheat model ( $Q_{\text{bio}}=0$ , Section 2.5.1). While this combination of assumptions is appropriate for isolating electrode-induced Joule heating for controlled comparative analysis, it warrants caution given the magnitude of temperature rises reported at the upper end of the investigated parameter range. For example, at ΔT≈30 °C, the temperature-independent, perfusion-free assumptions used throughout this study should not be considered physically defensible, since real tissue electrical and thermal conductivity, perfusion rate, and protein structure are all known to change substantially in this range. The parameter combinations exceeding this boundary (grid1–grid4 stress-test region, Section 2.7) are retained in Figure 7 for parameter-space completeness but are shaded to flag them as outside the model’s valid regime. These too should not be read as physically accurate absolute temperature predictions. Separately, the omission of perfusion-mediated cooling is expected to yield conservative upper-bound temperature estimates overall. However, this effect is unlikely to be strictly proportional across all electrode configurations, since perfusion-mediated cooling efficiency is depth- and temperature-dependent. Additionally, the spatial extent and depth of the electrothermal hotspot differ substantially between configurations. For example, the sparse grid1 configuration produces a more spatially concentrated superficial hotspot than the more distributed grid9–grid16 configurations (Section 3.1; Figure 10). As a result, the relative comparison between sparse and dense electrode configurations reported here may be influenced by differential, rather than uniform, perfusion-mediated cooling effects that were not captured in the present model. Neither temperature-dependent material properties nor perfusion effects were characterized through sensitivity analysis in the present study, but both represent important directions for future work.

Fourth, the globally reported current-density and temperature-rise metrics (Section 3.1) included a constant contribution from the return electrode that exceeds the stimulation array’s signal for denser configurations. For current density, the crossover occurred at N≳8 (Table 6). Since the return-electrode contribution was fixed across all seventeen configurations, it did not affect the relative ranking of configurations for N≤7 . As such, apparent plateau in both current density and ΔT for N≥8 –9 under the global evaluation domain reflects this fixed background contribution rather than a real plateau in the stimulation array behavior. This finding was validated directly through spatial decomposition of the full transient temperature field into stimulation, return, and global components (Section 3.1). The close match between the computed return contribution (1.38 °C) and the symmetry prediction (1.3695 °C) provides strong internal validation for both the decomposition strategy and the model symmetry.

Fifth, the present study did not include experimental thermal validation using tissue phantoms, infrared thermography, or controlled *in vivo* measurements. The reported electrothermal transition regions and parameter-space classifications should therefore not be interpreted as direct clinical safety limits, but rather as design considerations for HD neurostimulation systems.

Sixth, the equal-current allocation across contacts within each grid configuration (Section 2.3.2) reflects an assumed current-controlled device architecture. An alternative shared-terminal architecture would redistribute current across contacts according to local impedance and geometry rather than holding it equal, and was not simulated here because it requires a fundamentally different boundary-condition. The reported current-density and heating patterns, particularly for sparse configurations such as grid1, should therefore be understood as specific to the current-regulated architecture investigated, and may differ under a common-potential driving scheme.

Seventh, quantitative comparisons throughout this study rely on explicitly defined, scripted regions of interest (Section 2.8). This practice was adopted because interactive GUI-based region selection does not retain a persistent, auditable record of the exact spatial selection used to generate a given numerical result. This creates a risk that two evaluations carrying the same metric name could silently correspond to different underlying spatial domains. This risk is particularly relevant across separate analysis sessions or when comparing metrics computed by different contributors. This risk is likely to be general to finite-element post-processing workflows relying on interactive GUI-based region selection, rather than specific to the present software version. We recommend, as a methodological practice, that quantitative comparisons in electrothermal or similar finite-element modeling studies be based on exported, node-level solution data processed through explicitly defined and version-controlled region-of-interest definitions, to ensure that reported metrics remain traceable and reproducible.

Finally, the present simulations addressed a defined set of current-controlled pulsed stimulation conditions and focused exclusively on electrothermal behavior. Additional factors relevant to neurostimulation system performance, including neural activation efficiency, stimulation selectivity, comfort perception, and long-term tissue response, were not evaluated. The influence of individual electrode size and inter-electrode spacing on electrothermal behavior also remains to be investigated and represents an important direction for future work.

## 5 Conclusion

From a technical perspective, the present study provides a comparative computational framework to inform the future design of HD-inspired peripheral transcutaneous neurostimulation electrodes. Under the idealized modeling assumptions investigated here (i.e., homogeneous isotropic tissue properties, absent perfusion-mediated cooling, and idealized electrode-tissue interfacial contact), the electrode density and spatial distribution were shown to scale current distribution and tissue heating properties. Here, electrode count and total active electrode area were co-dependent governing factors (Section 3.2). As such, the development of such devices depends on the optimization of both characteristics. These findings represent qualitative, geometry-specific comparative modeling trends rather than validated thermal safety predictions, and should be interpreted within the scope of the idealized assumptions discussed in Section 4.3.

The work presented here will support future research in building HD neurostimulation systems, and *in vivo* human experiments that use HD neurostimulation grids for fine-motor control rehabilitation.

## Data Availability

The COMSOL Multiphysics model files and the parameter-sweep output datasets generated during the current study have been deposited in Zenodo and are available at https://doi.org/10.5281/zenodo.21284072.
